# Segmental duplications and evolutionary acquisition of UV damage response in the *SPATA31* gene family of primates and humans

**DOI:** 10.1186/s12864-017-3595-8

**Published:** 2017-03-06

**Authors:** Cemalettin Bekpen, Sven Künzel, Chen Xie, Muthukrishnan Eaaswarkhanth, Yen-Lung Lin, Omer Gokcumen, Cezmi A. Akdis, Diethard Tautz

**Affiliations:** 1 Max-Planck Institute for Evolutionary Biology, August-Thienemann Strasse 2, 24306 Plön, Germany; 2 Department of Biological Sciences, State University of New York at Buffalo, Buffalo, 14260-1300 NY USA; 3 Present address: Population Genomics and Genetic Epidemiology Unit, Dasman Diabetes Institute, P.O.Box 1180, Dasman, 15462 Kuwait; 4 Swiss Institute of Allergy and Asthma Research (SIAF), Davos, CH-7270 Switzerland

**Keywords:** Segmental Duplications, Core duplicons, SPATA31 gene family, Comparative Genomics, Copy number variation, UV response

## Abstract

**Background:**

Segmental duplications are an abundant source for novel gene functions and evolutionary adaptations. This mechanism of generating novelty was very active during the evolution of primates particularly in the human lineage. Here, we characterize the evolution and function of the *SPATA31* gene family (former designation *FAM75A*), which was previously shown to be among the gene families with the strongest signal of positive selection in hominoids. The mouse homologue for this gene family is a single copy gene expressed during spermatogenesis.

**Results:**

We show that in primates, the *SPATA31* gene duplicated into *SPATA31A* and *SPATA31C* types and broadened the expression into many tissues. Each type became further segmentally duplicated in the line towards humans with the largest number of full-length copies found for *SPATA31A* in humans. Copy number estimates of *SPATA31A* based on digital PCR show an average of 7.5 with a range of 5–11 copies per diploid genome among human individuals. The primate *SPATA31* genes also acquired new protein domains that suggest an involvement in UV response and DNA repair. We generated antibodies and show that the protein is re-localized from the nucleolus to the whole nucleus upon UV-irradiation suggesting a UV damage response. We used CRISPR/Cas mediated mutagenesis to knockout copies of the gene in human primary fibroblast cells. We find that cell lines with reduced functional copies as well as naturally occurring low copy number HFF cells show enhanced sensitivity towards UV-irradiation.

**Conclusion:**

The acquisition of new *SPATA31* protein functions and its broadening of expression may be related to the evolution of the diurnal life style in primates that required a higher UV tolerance. The increased segmental duplications in hominoids as well as its fast evolution suggest the acquisition of further specific functions particularly in humans.

**Electronic supplementary material:**

The online version of this article (doi:10.1186/s12864-017-3595-8) contains supplementary material, which is available to authorized users.

## Background

Gene duplications are a common source of evolutionary novelties [1]. Genome sequence analysis has shown that chromosomal fragments can become duplicated either in tandem or dispersed across chromosomes. The generic term “segmental duplications” has been coined for this form of duplication, and it is thought to have been particularly active in the primate lineage—especially in humans [2]. These duplications are associated with rapid structural changes, chromosomal instability and evolutionary rearrangements. The size of the duplicated regions ranges between one to several hundred kilobases. Approximately 430 delimited blocks of the human genome have been identified as regions for multiple duplications during hominoid evolution. In general, segmental duplications comprise about 5% of the human genome [2] and [3].

Some of the segmentally duplicated genomic regions are clustered around “core” duplication blocks (core duplicons) [4, 5]. The corresponding genes and gene families encoded by these core duplicons are different from classical segmentally duplicated gene families. Most of these core sequences show ubiquitous or global patterns of expression versus the ancestral locus [4]. Some of the most variable human CNV genes correspond to recently evolved gene families with embedded human core duplicons [6, 7]. Hence, it seems possible that gene families found with such core duplicons may be associated with specific adaptations during the evolution of the human lineage within the primate phylogeny [8–10]. However, only one of these recently duplicated gene families, *TBC1D3*, has been functionally characterized so far and was found to regulate EGF signaling [11–13].

We focus here on the *SPATA31* gene family (previously known as *FAM75A*), which is one of the fastest evolving gene families in the human lineage [8]. It belongs to the human core duplicon families [4], and it evolved from a single copy gene in mammals. *Spata31* in mice (previously known as *VAD1.3*) interacts with syntaxin and beta actin [14]. Knock-out mice are infertile due to a reduced number of sperm cells suggesting a role of the gene in mouse spermatogenesis [15]. We present here a comparative analysis between mice, old world monkeys (macaque) and hominoids (including human). We show that the *SPATA31* gene family expanded within the great apes by segmental duplication from one copy in mice to two copies in macaque and to multiple functional and non-functional copies along chromosome 9 in hominoid primates and humans. The *SPATA31* gene in primates acquired new upstream sequences that have led to broader expression and new protein domains suggesting an involvement in sensing and/or repairing UV damage. We provide experimental evidence in cell cultures that support this hypothesis.

## Results and discussion

### Gene duplication patterns

**Fig. 1:**
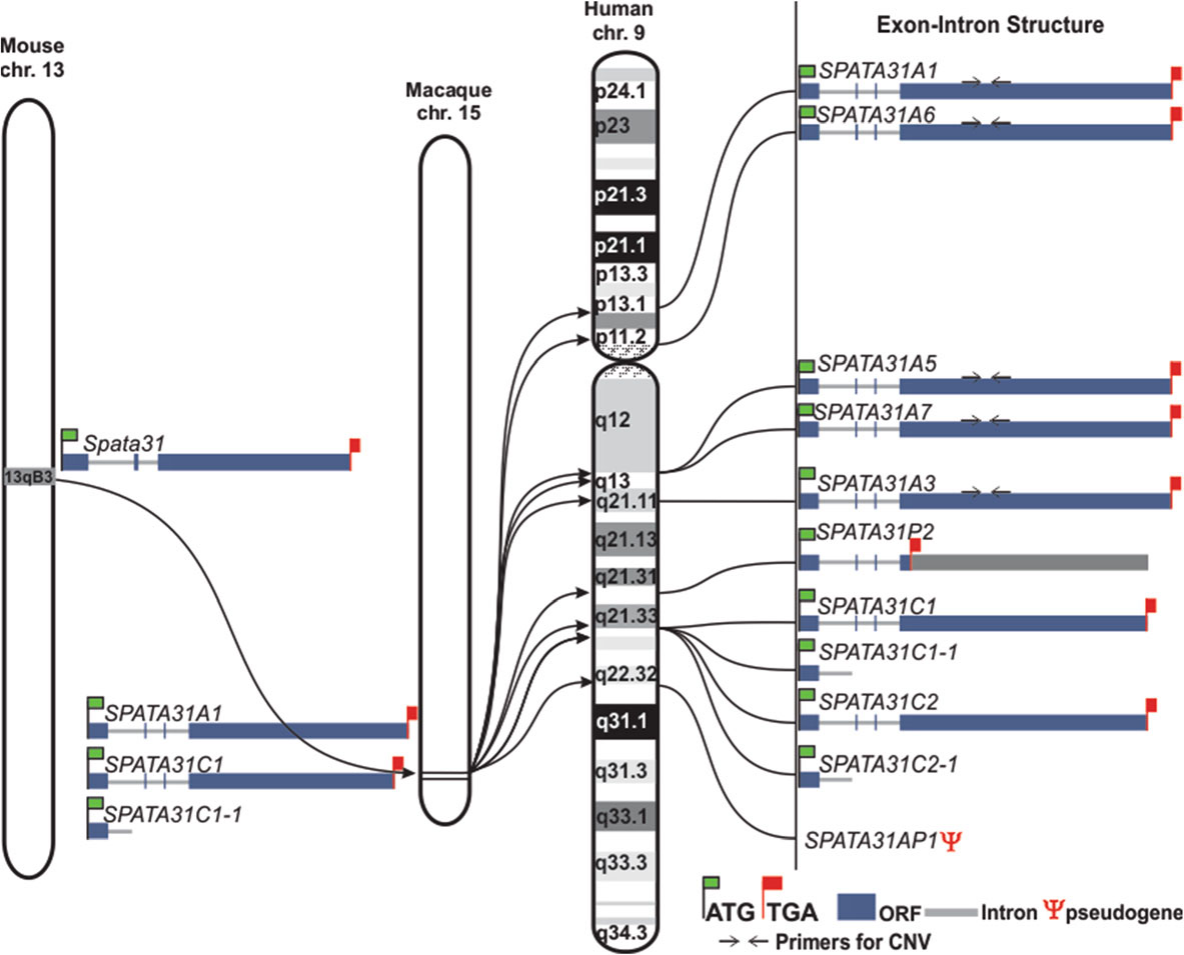
*SPATA31* gene family expansion in humans. The gene structure and chromosome localization of *SPATA31* genes based on the human reference (hg38), rhesus macaque (rheMac3) and mouse (mm10) genomes are shown. The mouse harbors a single copy that has duplicated and diverged into an A and a C type in macaque. Further segmental duplications are found in humans on chromosome 9

We conducted a detailed analysis of gene structure evolution and duplication patterns of *SPATA31* based on genome sequence comparisons. The mouse harbors a single copy of the *Spata31* gene on chromosome 13. In the macaque, there are two copies of *SPATA31*: type A and type C. Both expanded along chromosome 9 in humans (Fig. 1). There are other members of the gene family (*SPATA31D* and *E*) sharing a FAM75A domain, but they are otherwise much more diverged (Additional File 1) and are not considered further here.

Type A has seven segmental duplications in humans (numbers based on human genome build hg38), of which one is a clear pseudogene due to multiple stop codons (P1Ψ). Another encodes a truncated protein (P2) due to a frameshift mutation resulting in a premature stop codon in exon 4 (Fig. 1). Note that the annotation around *SPATA31A5* and *SPATA31A7* is uncertain because short non-sequenced regions interrupt the region. Type C has two copies in the human genome—each is preceded by a duplication of the first exon including the promoter sequence (Fig. 1). However, these additional promoters do not appear to initiate transcripts. The gene lengths and the protein coding regions of *SPATA31* genes differ between A and C types. The predicted molecular weights are 157 kD for the A type (A1) and 130 kD for the C type (C1).

To trace the expansion of the family, we assessed copy numbers in fully sequenced genomes of sequenced individuals from macaque, orangutan, chimps and twelve humans including Us_Ishim, Denisovan and Neandertal. The results show that there was on average a progressive increase of segmental duplications of the *SPATA31* gene locus towards humans (Additional File 2).

**Fig. 2:**
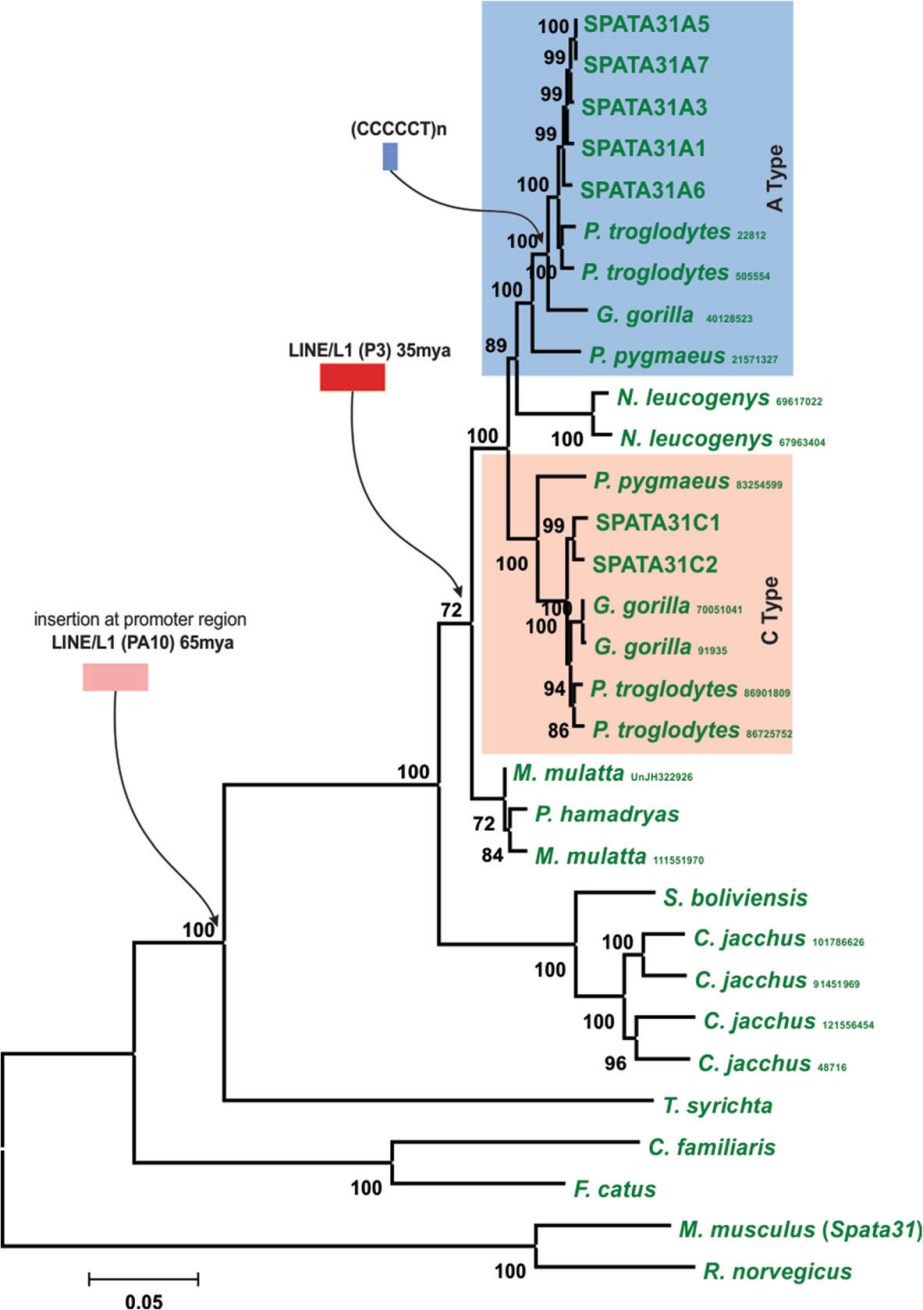
Evolutionary emergence of the human *SPATA31* gene promoter structures. Phylogenetic reconstruction of full length SPATA31 proteins (exon4 - longest coding exon) in different primates, cat, dog, rat and mouse species using the NJ method [43] with bootstrap values (500 replicates) indicated at the branches [44]. Species names are: rat (*R. norvegicus*), mouse (*Mus musculus domesticus*), cat (*F. catus*), dog (*C. familiaris*), tarsius (*T. syrichta*), marmoset (*C. jacchus*), saimiri (*S. boliviensis*), baboon (*P. hamadryas*), rhesus macaque (*M. mulatta*), gibbon (*N. leuco*genys), orangutan (*P. pygmaeus*), gorilla (*G. gorilla*), chimpanzee (*P. troglodytes*) and human (*H. sapiens*). Repetitive elements *LINE/L1-PA10* (pink), *LINE/L1-P3* (red) and (CCCCCT)n (blue) that were found within the *SPATA31A* promoter region are indicated with an arrow to show approximate integration time

A detailed comparison of the promoter regions including *LINE/L1* (MD and MER31A) elements revealed that there are two different general promoter structures shared by all *SPATA31A* and *SPATA31C* genes respectively (Fig. 2 and Additional File 3). In particular, the promoter region of *SPATA31C* was subjected to multiple rounds of rearrangement resulting in a composite promoter structure consisting of three *LINE/L1*, MD, MER31A, PA10, three AluY and one ERV1 elements. In contrast, the functional *SPATA31A* promoters are composed of LINE/L1-P3 and PA10 retroviral elements (Additional File 3). The main expansion of the PA10 element occurred about 65 Mya and the expansion of the P3 element occurred about 35 Mya (reviewed in [16] and [17]). Accordingly, no P3 element is detected in the promoter regions of New World Monkeys. An insertion of a CCCCCT simple repeat is observed in gorillas, chimpanzees and humans at the time where the main expansion of the family is observed. Thus, we propose that the promoter region of *SPATA31A* was restructured in a stepwise manner by integration of LINE and a CCCCCT simple repeat within the primate phylogeny (Fig. 2).

Fig. 2 also includes phylogenetic comparisons of the A and C-type copies in humans and chimpanzees. The respective duplicated copies for each type are more similar within each species than between the two species. This is a clear sign of concerted evolution of the gene family within each species [18]. In case of segmentally duplicated genes, this would occur most likely by frequent gene conversion events such that the duplicated copies retain higher similarity within their evolutionary lineage.

### Protein domain evolution

**Fig. 3:**
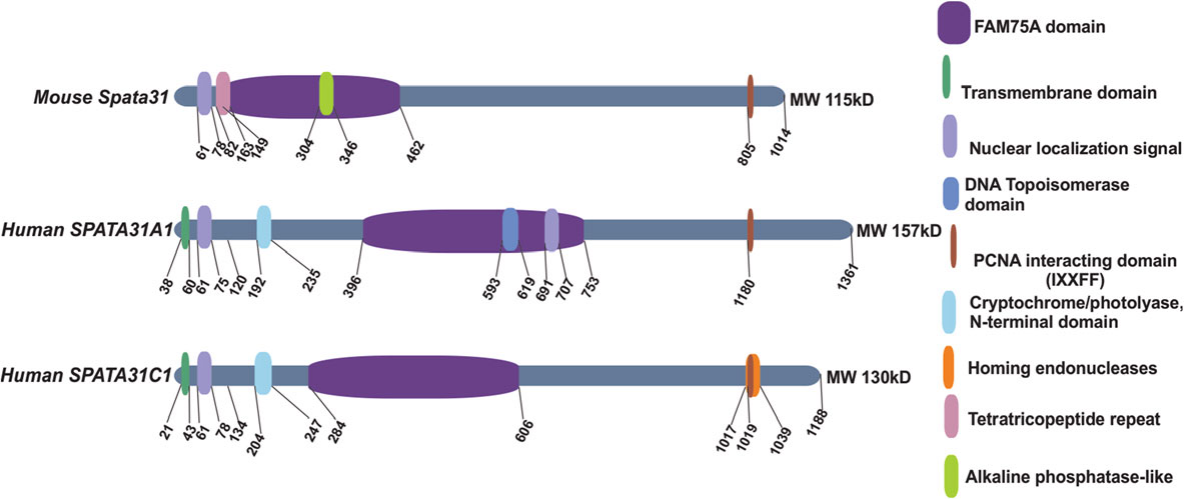
Domain patterns of SPATA31 proteins. Protein domain patterns of mouse *Spata31* (NM_030047.2), human *SPATA31A1* (NM_001085452) and human *SPATA31C1* (NM001145124) based on comparative genomics, smart protein database (http://smart.embl-heidelberg.de/) and motif search (http://myhits.isb-sib.ch/cgi-bin/motif_scan). Protein domains are depicted with colored boxes (right). Note that the human domain structure is the same as in the other primates including macaque. See Additional File 5 for details of the domain descriptions and definitions

Motif scans and multiple alignment analysis (Additional File 4) predict that there are several domain structures present in SPATA31 proteins and that they differ between the subtypes and between primates (represented by human) and mouse (Fig. 3; Additional File 5). All share a FAM75A domain in the middle, a nuclear localization signal in the N-terminal part of the protein and a PCNA-interacting domain at the C-terminus. The primate genes have a cryptochrome/photolyase domain and a proline rich region. SPATA31A has a DNA topoisomerase domain and a further nuclear localization signal in the middle of the protein. The mouse SPATA31 protein has an alkaline phosphatase and a TRR-like domain that is not found in the primate proteins (Fig. 3). The *SPATA31* gene family also shows similarities to Epstein Barr Virus (EBV)–BPLF1 and CRY2 proteins (Additional File 6), and we found through antibody staining (see below) partial co-localization with CRY2 protein (Additional File 7). CRY2 is one of the circadian clock proteins involved in blue light-dependent regulation of the circadian feedback loop [19]. Cryptochromes play an important role in intrinsic apoptosis induced by UV mimetic and radiometric compounds [20]. EBV-BPLF1 protein has been implicated to interact with PCNA and to delay the DNA trans-lesion synthesis (TLS) repair mechanism [21]. The TLS repair mechanism was also shown to be important during UV irradiation-induced DNA damage repair [22]. Hence, the N-terminal region of the SPATA31 proteins acquired several important functional domains compatible with the acquisition of an UV response when compared to the mouse SPATA31 proteins.

### Copy number variation of *SPATA31A*

**Fig. 4:**
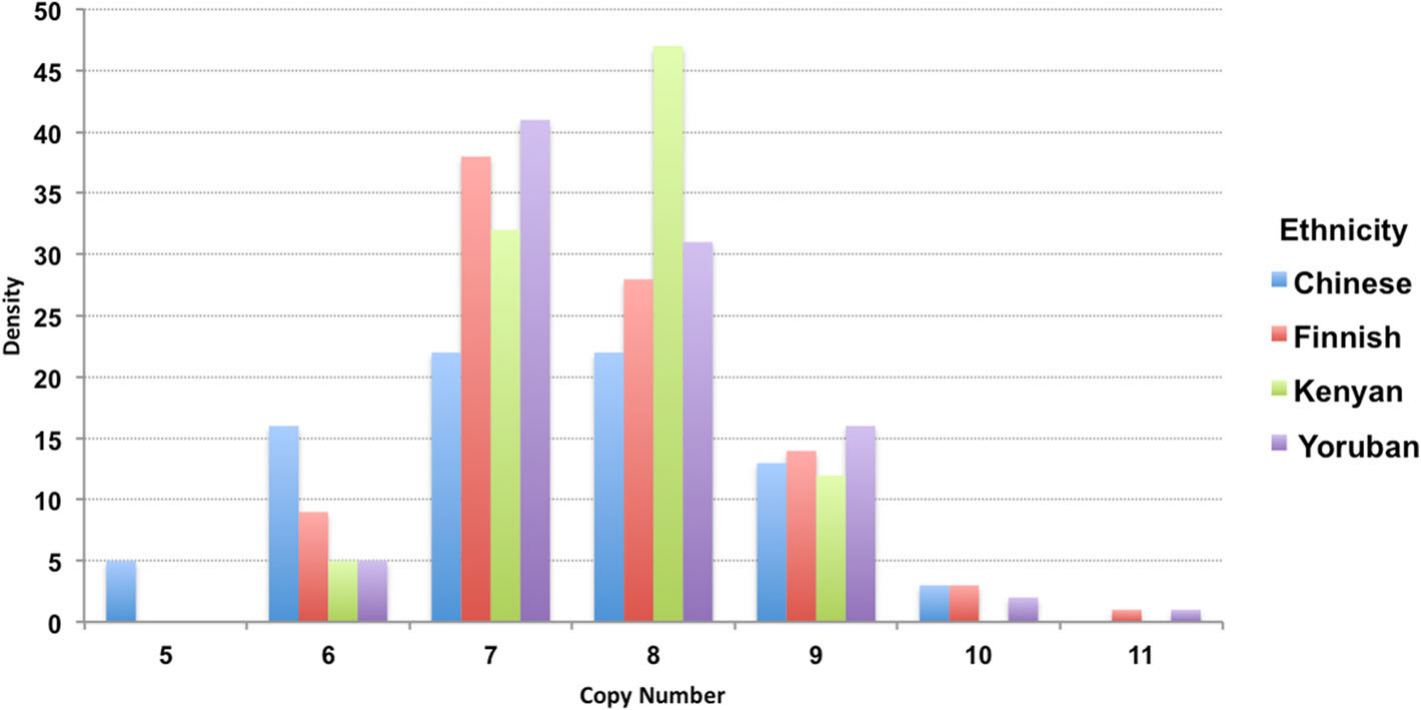
Copy number variation of *SPATA31A* in human populations. Copy numbers of individuals were estimated based on digital PCR and then binned into number classes (see Additional File 15 for detailed data)

To determine copy number variation for the *SPATA31A* genes in human populations we used genomic DNA panels for a subset of individuals that were also used in the 1,000 Genomes Project. We genotyped 322 samples from the MGP00001 (Finnish in Finland), MGP00002 (Han Chinese South), MGP00008 (Luhya in Webuye, Kenya) and MGP00013 (Yoruba in Ibadan, Nigeria) panels from the NHGRI Repository at Cornell using digital PCR with *SPATA31A*-specific primers. We found on average around 7.5 copies per diploid genome, with a range between 4.5 to 10.8 copies on the extremes (Fig. 4). There were no obvious differences between the means of each population, but there were differences in the breadth of distribution with the highest in the Chinese population (mean and standard deviation for Chinese (7.24 and 1.24), Finnish (7.61 and 0.98), Kenyan (7.62 and 0.71), Yoruban (7.62 and 0.85)) (Fig. 4).

### RNA and protein expression

**Fig. 5:**
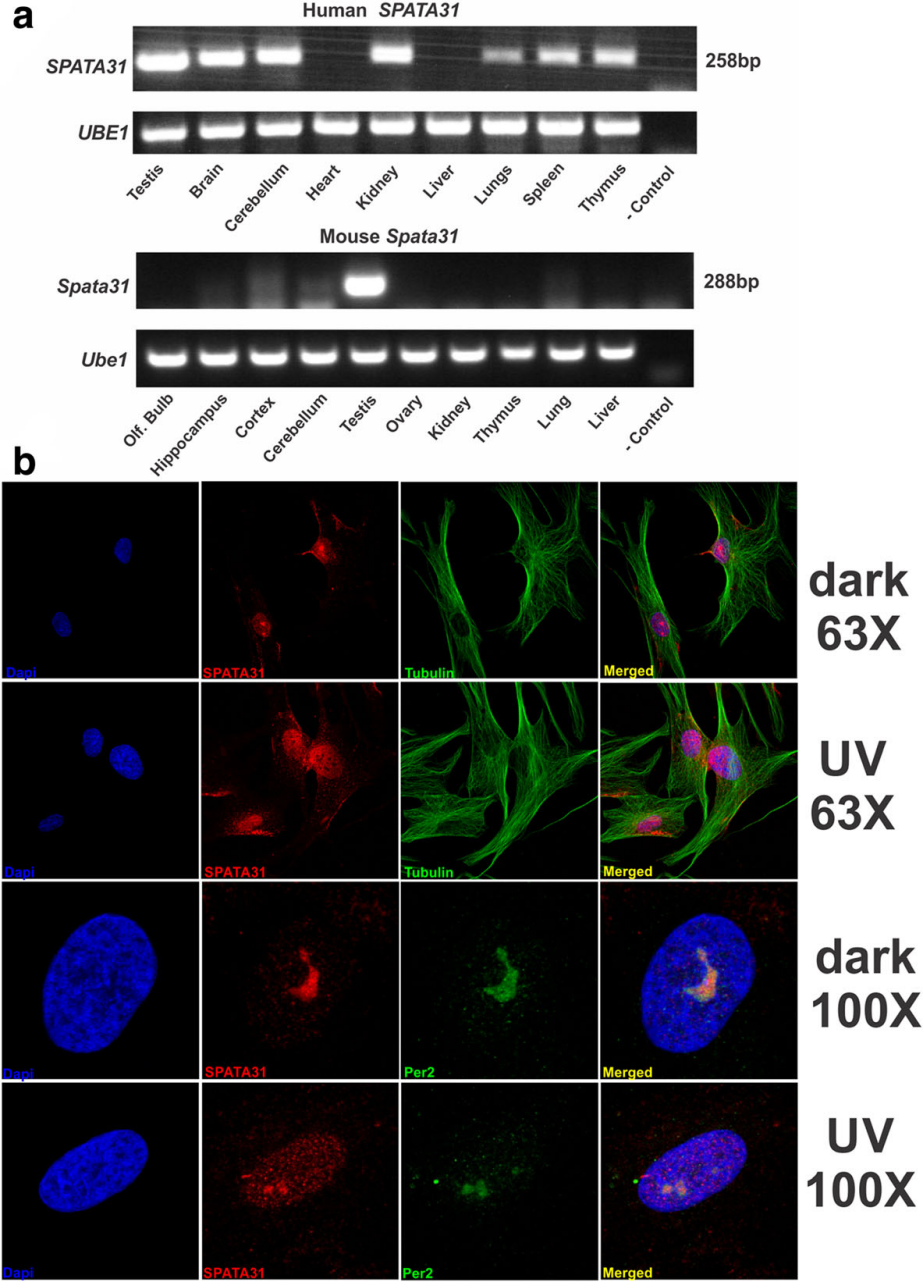
Expression analysis of *SPATA31* RNA and protein within human primary fibroblast (HFF) cells. **a** RT-PCR analysis on cDNA isolated from total human RNA (Clontech) and from Mouse RNA. The PCR primers were designed to amplify the highly conserved region within the long coding exon for human. For mouse, we amplified a region spanning exons 1–3. The UBE1 gene was used as control. Quantitative PCR for the same tissue samples is shown in Additional File 8. **b** Antibody staining of HFF cells with fixation under dark conditions versus a treatment with 200 J/m^2^ UVC and 24 h further growth at two different magnifications (63x and 100x). Nuclei in the first column are stained with DAPI (blue), and SPATA31 staining is shown in the second column (red). In the third column, the green staining in the 63x pictures is cellular cytoskeleton detected with an antibody against tubulin and in the 100x columns the nucleolus with an antibody against Per2 [45]. See Additional File 12 for further quantification of SPATA31 response of re-localization upon different exposure to UVC. We noted some variation of relative SPATA31 protein localization between cytoplasm and the nucleus depending on the cell cycle and fixation protocol as well as dark–light conditions during fixation similar to other UV response proteins such as H2Ax (reviewed in [46] and see Methods)

We used RT-PCR to assess the expression of *SPATA31* (A and C type combined) in different mouse and human tissues. We found that the mouse expresses the gene only in the testis, while humans show expression in multiple tissues (Fig. 5a). Such an expansion of expression into other tissue types was also seen for the segmentally duplicated *Morpheus* [23] and *LRRC37* genes [10], [24] and in primates [25]. The differences in expression may be associated with the observed restructuring of the *SPATA31A/C* gene and the promoter region by repetitive elements during the evolution of primates (see above). However, quantitative PCR showed that even in humans the highest level of expression is still in the testis—expression in other tissues is still quite low (Additional File 8).

We raised an antibody against peptides shared by the A and C types to assess the protein localization at the sub-cellular level (see Additional File 9 for documentation of the specificity of the antibody). We found that during mitosis the SPATA31 protein accumulates around the spindle (Additional File 10). In lung and sinus tissue, the protein is mostly expressed in the epithelial layer, but almost all of the cells show expression in tonsil tissue (Additional File 11). We focused most of the further analysis on human foreskin fibroblasts (HFF), which represent primary and non-immortalized cells of the ectoderm. Here, SPATA31 proteins are primarily localized to the nucleus with an enhanced staining seen in the nucleolus—especially under dark conditions (Fig. 5b).

Based on the domain and similarity analyses above, we reasoned that SPATA31 protein may be involved directly or indirectly in the repair pathway of UV-induced DNA damage and/or for the recruitment of the DNA repair molecules to damaged sites via its PCNA interaction domain. Therefore, we exposed various human cell lines to different strength and time intervals of UVC light. We found a consistent shift and/or upregulation from nucleolar localization to a spread across the entire nucleus in these experiments (Fig. 5b and Additional File 12). This effect was also seen in other proteins involved in UV damage repair [26].

### *SPATA31* function

To investigate the molecular function of *SPATA31A/C* genes, we targeted exon 1 of the *SPATA31A/C* genes via CRISPR/Cas mediated mutagenesis [27, 28] (Additional File 13) in human foreskin fibroblast cells (HFF). We did not expect to obtain a full knockout because we were targeting a multi-copy gene; rather, the goal was a reduction in functional gene numbers. To estimate the types and frequencies of mutations induced across different copies, we amplified fragments around the expected lesion and sequenced them via Illumina sequencing. This allowed us to identify single cell clones with low and high frame shift mutation numbers (Additional File 14). For further analysis, we selected one of the best growing cell lines from each class, Cl1 (low number of frameshifts) and Cl2 (high number of frameshifts) using untreated HFF cells as control.

**Fig. 6:**
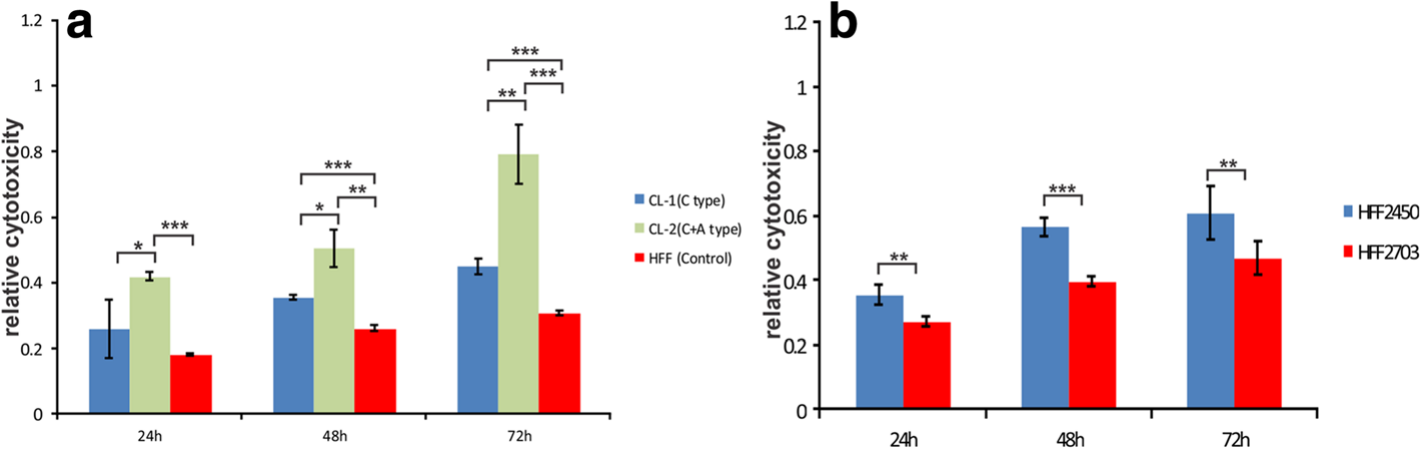
Increased UV sensitivity of mutated HFF cells. To assess the UVC sensitivity, we used the LDH cytotoxicity assay to measure the release of LDH from damaged or dead cells. This is a very sensitive cell toxicity test [37] and [38]. **a** Differences between controls and mutated cells between the two types of mutated cell lines, Cl1 (low) and Cl2 (high) and non-mutated control cells. **b** Differences between the low copy cell line HFF2450 and the high copy cell line HFF2703. *P*-values of Student’s *t* test are indicated as * < 0.05, ** < 0.01, and *** < 0.001

We tested whether the mutated cell lines would show an effect with respect to UV-induced cell damage and death. Both the Cl1 and the Cl2 cells had elevated sensitivity to UVC treatment compared to control. Stronger effects were seen in the Cl2 cells (Fig. 6a). Using digital PCR analysis we found that the two different HFF cell lines had incidentally a natural difference in copy number. Cell line HF2450 has eight copies of *SPATA31A* and three of *SPATA31C*; cell line HF2703 has nine *SPATA31A* copies and four *SPATA31C* (numbers refer to diploid copy number). We compared these two cell lines in the same UV damage test and found that the one with more copies is somewhat less sensitive to UVC irradiation (Fig. 6b).

## Conclusions

The spermatogenesis phenotype of *Spata31* knockout in mice [15] suggests that the ancestral function of *SPATA31* is in the pathway of sperm formation. It is currently unknown whether it retained this function in humans, but the high expression in human testis points to an involvement in spermatogenesis as well. However, *SPATA31* has clearly also acquired new functions in the primate lineage. Its acquisition of a cryptochrome/photolyase domain may allow it to sense UV light, although photolyase domains are so far only known from circadian clock functions where they sense blue light [29]. Further, it may recruit other DNA repair genes through its topoisomerase domain and the PCNA interacting motif by opening the chromatin structure.

Given that increasing exposure to UV light would have played a role in primate and hominoid diurnal evolution, it is likely that the original acquisition of new functions is connected to the increased exposure to sunlight. The further expansion into multiple copies in the line towards humans may have had the same reason. Humans in particular got exposed to more UV light in conjunction with loosing their body hair. But this would have been dependent on skin color and the parts of the world where they lived. This could explain why there is still copy number variation at this locus. However, these explanations remain necessarily speculative and require deeper population analysis in humans as well as further functional studies.

## Methods

### Nomenclature

The naming of the paralogous SPATA31 variants follows the Hugo Gene Nomenclatures (HGNC) (http://www.genenames.org). The corresponding gene names are also implemented in the GRCh38/hg38 reference assembly of the human genome [30].

### RT-PCR

Total RNA used for cDNA preparation was extracted from mouse tissues using the RNeasy kit (Qiagen). Total RNA from human tissues was purchased from Clontech (Cat No: 636643). Polyadenylated mRNA was isolated using the Oligotex mRNA mini kit (Qiagen). cDNA was prepared using the Reverse Transcriptase PCR kit (Fermentas) according to manufacturers recommendation with the exception of: Odt and random hexamer primers were added at equal concentration and the reaction mixture was incubated for 60 min and immediately used for subsequent RT-PCR or RACE-PCR. *UBE1* was used as positive control. PCR was performed in 20 μL reactions composed of 0.8 μL of a 10 μM dilution of the forward primer and reverse primer, 10 μL of PCR Master Mix (Roche −11636103001). See Additional File 16 for the PCR conditions and primer sequences.

### Quantitative real-time PCR

*SPATA31* transcripts were analyzed by a quantitative PCR assay using the ABI SYBR Green System (Applied Biosystems 7500 Real Time PCR System) with primers directed against the last coding exon (exon 4). The amount of measured transcripts was normalized to the amount of the *Ef1alpha* transcript. See Additional File 16 for the real-time PCR conditions.

### 5’RACE-PCR

Single-stranded cDNA (described above) was purified by using a rapid PCR purification kit (Roche). The terminal deoxynucleotidyl transferase (TdT) reaction was prepared as follows: 16.5 μL cDNA, 5 μL TdT + Reaction buffer (Amersham), 2.5 μL dCTP (2 mM) were incubated for 3 min at 94 °C, 1 μL of TdT was added and incubated for 15 min at 37 °C, followed by an inactivation step for 5 min at 65 °C. PCR was performed on the cDNA tagged with polyC using the primer *5’Anc* and *SPATA31_R*. PCR products were purified using the rapid PCR purification kit (Roche) and a second round of nested PCR was performed. The resulting PCR products were cloned into the PGEM-T easy vector system (Promega) and insert sequences were determined by Sanger based sequencing.

### Other DNA Methods

All multiple sequence alignments were generated using ClustalW [31, 32] Phylogenetic trees were generated using MEGA [33] using the Kimura 2-parameter model [34]. All positions containing alignment gaps and missing data were eliminated only in pairwise sequence comparisons (pairwise deletion option). NextGen sequencing was done on Illumina MiSeq. The resulting sequencing reads were first quality checked, PCR duplicates were removed and then mapped by bwa mem [35] and the mapped reads were visualized by IGV 2.3.55 [36]. Reads with frameshift mutations around the CRISPR/Cas target site were visually identified and counted.

### Cell culture

HFF cells (HFF2703 (CRL-2703) and HFF2450 (CRL-2450) purchased from ATCC) were grown in IMDM (Cat No: 21980–065, Life Technologies, Paisley, U.K.; GIBCO) and CV1, and L929 cells were grown in DMEM (Cat No:41966–29, Life Technologies, Paisley, U.K.; GIBCO) supplemented with 10% (vol/vol) FBS (PACBIO) and 100U penicillin-streptomycin (Life Technologies; GIBCO). The culture was incubated at 37 °C including 5% CO_2_.

### Western blot analysis

Proteins were run on SDS-PAGE gels and transferred to nitrocellulose membrane by electroblotting. Ponceau-S (0.1% Ponceau-S (w/v) (Sigma), in 5% acetic acid) staining was used to identify the location of the proteins on a PVDF membrane. The membrane was blocked with 5% milk powder or BSA, 0.1% Tween 20 (Sigma-Aldrich), for 15 h at 4 °C. Antiserum/antibody was diluted in PBS, 5% milk powder or BSA, 0.1% Tween-20, and protein bands were visualized using the enhanced chemiluminescence (ECL) substrate kit (Amersham) and X-ray film sheets.

### CRISPR/Cas targeting vector preparation

10 μg of pX260 or pX330 were digested with *BbsI* (NEB) for 3 h at 37 **°**C. Digested pX260 or pX330 vectors were purified by QIAEXII Extraction Kit (Qiagen) according to manufacturers recommendation. Targeting guide RNA sequence specific to exon1 of *SPATA31* genes was designed by using the CRISPR design tool (crispr.mit.edu). Among 32 possible targeting guide RNAs, “Fm_ex1_(GATATCCACACCCATGGTG)” was selected as a guide RNA to avoid off-target possibilities. Among 98 possible target sides of Fm_ex_1, based on the CRISPR Design tool, there were only three off-targets within the exonic region outside of *SPATA31* genes with very low targeting score (less than 0.2). Complementary oligo-nucleotides representing the target sequence were annealed and phosphorylated according to [27]. The ligation reaction was treated for 30 min with PlasmidSafe exonuclease (Epicenter) to prevent unwanted recombination products after the ligation reaction (Quick ligation kit (NEB)) of purified vector and phosphorylated and annealed oligos. 5 μL of the ligation reaction was transformed to Top10 or Stbl3 competent cells (Life Technologies; GIBCO). Positive clones were verified by sequencing and DNA was isolated using the Maxiprep plasmid DNA preparation kit (Qiagen).

### Transfection of cells

Cells were seeded on T75 cm tissue culture flasks (Thermo Fisher Scientific) before the transfection and incubated overnight. Amaxa Nucleofactor (Lonza) programs for the transfection of the cells were optimized according to manufacturers recommendation. We found that program A033 is the best for most of the cells and, therefore, all the transfections were performed using the program A033. HFF cells (CRL-2703, ATCC) are transfected with the mock and px330-FMex1 alone or together with px260-FMex1 constructs by using Amaxa Basic Nucleofactor Kit for Primary Mammalian Epithelial Cells (Cat No. VPI-1005). Cells were either directly subcloned or subcloned after selection with puromycin in mouse embryonic fibroblast (MEF) pre-seeded feeder cell 96 well plates in a dilution ratio of 1 cell per well in 100 μL of IMDM medium ((Cat No: 21980–065, GIBCO) 3 days after transfection. After approximately 5–6 weeks individual cell clones identified by colony formation in the 96 well plates were successively subcultured to 24, 48 and 6 well plates.

### Antibody generation for Spata31 protein

Rabbit polyclonal antisera were raised against the peptide (CHKSEKSRKPNLEKHE) located at the C-terminal region of SPATA31 protein using keyhole limpet hemocyanin (KLH) as a carrier protein. The peptide synthesis (more than 80% purity) and injections to two rabbits were performed by Pocono Rabbit Farm and Laboratory (USA). Antibody titers were determined by an ELISA assay and the specificity of the rabbit polyclonal antibody against SPATA31 protein was tested both in western blot and immunofluorescence analysis (see Additional File 9). 10 mL of 2^nd^ and 3^rd^ bleeds were purified against the peptide by affinity purification (Affigel - BioRad).

### LDH Cytotoxicity assay

We used the LDH cytotoxicity assay (Thermo Scientific Pierce) to quantitatively measure lactate dehydrogenase (LDH) released into the media from damaged cells as a biomarker for cytotoxicity according to manufacturers recommendations [37] and [38]. Equal amount of cells (about 1 million) were plated on a 5 cm plate. After 2 days of incubation at 37 **°**C in a humidified incubator supplemented with 5% CO_2_, the medium was removed and cells were immediately exposed to 200 J/m^2^ UVC light (Hoefer UVC 500-UV cross linker machine (Amersham)). After the UV treatment fresh medium was added and cells were incubated for 24 h. Subsequently, after each 24 h of intervals (24, 48 and 72 h) 1 ml aliquots of medium were taken from each plate and frozen at−20 **°**C until the LDH cytotoxicity assay was performed. The remaining medium was removed and fresh medium was added for additional intervals. For the comparison of mutation and natural CNV variations, experiments repeated in two and three independent replicates, respectively. Three replicates of 50 μL of growth media (IMDM (see above)) taken from each sample at the indicated time points were used according to the LDH cytotoxicity assay kit protocol and absorbance measurement (490–600 nm) was performed on a NanoQuant infinite M200PRO (Tecan) using Magellan v7.0 software.

### Immunofluorescence analysis

Cell lines for immunofluorescence analysis were grown in 24-well plates including previously added cover slips to each well. The growing media were removed and the cells (either treated or transfected) were directly fixed with 0.5 mL of −20 °C cold methanol or PBS/1.5% paraformaldehyde (PFA) for 10 min at room temperature (RT) followed by−20 °C cold methanol for 10 min at −20 °C. Cells were washed three times with PBS and additionally washed with 1 mL of PBS/0.1% saponin (Sigma-Aldrich) by incubating for 20 min at RT on a shaker in slow motion (50 rpm). The wash buffer was removed and cells were immediately blocked by adding PBS/0.1% saponin/3% BSA (bovine serum albumin, fraction V, Sigma Aldrich) and incubated for 1 h at RT in 24-well plates. Coverslips were incubated with 0.25 mL of PBS/0.1% saponin in a humified environment for 1 h at RT or overnight at 4 °C. Cells were washed 3× with 1 mL of PBS/0.1% saponin. After washing, coverslips were incubated with the appropriate secondary antibody (Alexa Fluor® 488, 546 or 594 (Molecular Probes, Life Technologies; GIBCO)) dilutions (1:2000) in a humidified environment for 1 h at RT in the dark. Cells were washed 3× with 1 mL of PBS/0.1% saponin for 20 min at RT on a shaker in slow motion (50 rpm). Finally, coverslips were put onto a microscope slide with 10 μL of ProLong® Gold Antifade Mountant, which contains DAPI (Cat No: P36941, Molecular Probes, Life Technologies; GIBCO). After overnight incubation, cells were observed with a Leica (DM5000) confocal fluorescence microscope, using the Leica software (Leica Application Suite LAS X) for photography and analysis. Of note, we noticed a slight variation in the subcellular localization of SPATA31 proteins depending on treatment conditions. First, SPATA31 is very sensitive to light exposure and we needed to keep the cells in the dark and fix them very fast for the UV response experiments. Second, when methanol at −20 °C was used for the initial fixation, most of the immunofluorescence signal was detected in the nucleus, whereas when we fixed the cells only with 4% PFA the signal was seen both at the cytoplasmic membrane and the nucleus. Methanol is known to solubilize membrane bound proteins, i.e. this may have caused the loss of membrane signal under the methanol fixation conditions. Therefore, we prefer to use initial fixation of PBS/1.5% paraformaldehyde (PFA) for 10 min at room temperature (RT) followed by −20 °C cold methanol for 10 min at −20 °C.

### DIGITAL PCR for copy number detection

The human 1000 genome sample data were used according to the Fort Lauderdale Agreement, January 2003 (http://www.1000genomes.org/data#DataUse). We used the genomic DNA panels for a subset of individuals that were also used in the 1000 Genomes Project. Specifically, we genotyped a total of 366 samples from the MGP00001 (Finnish in Finland), MGP00002 (Han Chinese South), MGP00008 (Luhya in Webuye, Kenya) and MGP00013 (Yoruba in Ibadan, Nigeria) panels from the NHGRI Repository at Coriell. The sample names are listed in Additional File 15 along with the estimated copy number states for these genomes. PCR reaction mixtures were prepared from 10 μL of 2x ddPCR Supermix for Probes (Bio-Rad, Hercules, CA, USA) mixed with HindIII restriction enzyme, 1 μL of the EIF2C1 primers with a fluorescent labeled probe (1 μL of the *SPATA31* primers with a fluorescent labeled probe), 1 ng of DNA template and 6 μL of molecular grade water to make a 20 μL final volume (see Additional File 16 for the primer and probe list). This reaction mixture was prepared in an Eppendorf 96-well twin.tec PCR plate and then loaded into the Automated Droplet Generator (Bio-Rad, Hercules, CA, USA) to generate oil droplets in each well of the plate containing 20 μL of the reaction mixture. After droplets were generated, the plate was sealed with a pierceable foil heat seal using PX1™ PCR Plate Sealer (Bio-Rad, Hercules, CA, USA) and then placed on a thermal cycler for amplification. Thermal cycling conditions were as follows: 95 °C for 10 min (1 cycle), 94 °C for 30 s (ramp rate 2.5 °C/s) and 56 °C for 60 s (ramp rate 2.5 °C/s) (40 cycles), 98 °C for 10 min (1 cycle), and 12 °C hold. After PCR, the 96-well PCR plate was loaded on the QX100™ Droplet Reader (Bio-Rad, Hercules, CA, USA), which reads the droplets from each well of the plate. The data obtained were analyzed using QuantaSoft™ analysis software provided with the QX100™ Droplet Reader. We scored the copy numbers by measuring the concentration of the target, *SPATA31,* relative to the concentration of the reference for population analysis, *EIF2C1*.

### In silico estimation of *SPATA31* copy number in different lineages of primates

To estimate the copy number of *SPATA31* sequences across different primate genomes, we utilized whole genome sequencing data of available genomes. Specifically, we analyzed nine modern humans genomes (from the 1000 Genomes Project) from different ethnicities. We also downloaded the genome data of the 45,000 year old modern human - Ust_Ishim from Siberia [39]. In addition, we compiled data from Denisovan [40], Altai Neandertal [41] genomes both of which have high read-depth as compared to most ancient genomes. For nonhuman primates, we used data from Gokcumen at al., [25], which includes five chimpanzees, five orangutans, five rhesus monkey genomes. We used these data to record the read depth of the *SPATA31* homologous sequences in these genomes (samtools v1.3) [42]. Primate reference genomes do not reflect the full scope of copy number of *SPATA31*, i.e., some of the *SPATA31* sequences may not be represented in the reference genomes. We surmised that even if there is one copy of the *SPATA31* sequence, the reads from other *SPATA31* sequences will map to that reference location. Based on this we summed up the total read-depth and normalized the resulting read-depth with the overall read-depth observed in the specific genome. This pipeline allowed us to comparatively estimate the total copy number of *SPATA31* sequences across and within species (Additional File 2).

## Additional files

Additional file 1: Phylogeny of SPATA31 proteins. Neighbor-Joining tree [43] of SPATA31 protein sequences from human (capital letters) and mouse (small letters); bootstrap values (1000 replicates) are shown next to the branches [44]. The tree includes the distant gene families D and E. (PDF 362 kb)
Additional file 2: Copy number estimates of *SPATA31* in different lineages of primates. For each genome, the read depth at all putative *SPATA31* sites were recorded from the primary genome sequence reads, summed up, and normalized by genome-wide read depth to obtain the haploid copy number estimation (y-axis). The genome samples analyzed are listed on the x-axis, including a Denisovan, a Neandertal, a Ust_Ishim, nine modern humans, five chimpanzees, five orangutans, five rhesus monkeys. Samples are color coded according to their species. Individual ancient genomes were also color-coded. The color-coding is shown on the right of the plot. See Methods for the details of the *SPATA31* copy number estimates in primates. (PDF 150 kb)
Additional file 3: Promoter comparisons between mouse *Spata31* and human *SPATA31A/C* genes. Overall schematic comparison with the experimentally determined transcriptional start sites indicated with respect to the ATG start codon. To identify the transcription initiation sites, we performed 5’RACE (rapid amplification of cDNA ends). This yielded an initiation site 38 bp upstream of the start codon for *SPATA31A*, which is consistent with the canonical start site present in sequence databases (note: it differs by the addition of only 18 additional bp when compared to the reference mRNA sequence (NM_001085452) for *SPATA31A1*. Compared to mouse, we found that LINE/L1 and other elements inserted into the promoter region of *SPATA31* genes, creating different upstream regions for A and C types. Further, the type A genes acquired a (CCCCCT)n simple repeat which provides potential Sp1 binding sites, present within the promoter region, which can be traced to the progenitor of humans, gorillas and chimpanzees (compare Fig. 2). (PDF 393 kb)
Additional file 4: Alignment of SPATA31 proteins. Alignment of mouse and human SPATA31A/C proteins by Clustal-W [31] and [32]. BoxShade server (http://www.ch.embnet.org/software/BOX_form.html) was used to highlight similar (grey) and identical (black) amino acids. Identified domains corresponding to the ones shown in Fig. 3 are indicated. (PDF 126 kb)
Additional file 5: Table with details of domains found in SPATA31 proteins. (XLSX 9 kb)
Additional file 6: Alignment of SPATA31 protein with CRY2 and EBV-BPLF1 proteins. The figure shows the alignment of SPATA31A1 (Q5TZJ5-1) protein with CRY2 (Q49AN0-1) (Top) and ENV-BPLF1 (P03186) (bottom) proteins. Similar (gray) and identical (black) amino acids are highlighted by using boxshade (http://www.ch.embnet.org/software/BOX_form.html). Alignment was performed by using Clustal-w and manually edited and only the region showing highest homology/similarity region is shown (starting from the amino acid position 166 of SPATA31A1) [31]. The overall calculated identity and similartiy between SPATA31A1 and CRY2 protein within the given window of alignment is 15% and 23%, and between SPATA31A1 and EBV-BPLF1 protein is 17% and 28%, respectively. Identity and similarity was calculated by GeneDoc software (version 2.7.000). (PDF 53 kb)
Additional file 7: Co-localization of *SPATA31* with CRY2 protein in HFF cell line. Immunofluorescence analysis of SPATA31 protein in HFF cells showing the co-localization of the SPATA31 protein with CRY2 protein. Cells were stained with a 1:100 dilution of SPATA31 antibody (red) and CRY2 antibody (Abcam, 1;250) (green) and merged (yellow). The nucleus is visualized by DAPI staining (blue). Note that these cells had been exposed to light and not syncronized, i.e. the nucleolar staining is not so prominent. (PDF 1403 kb)
Additional file 8: Quantitative expression analysis of the *SPATA31* gene family in human. Quantitative real time PCR analysis on Human *SPATA31* genes based on cDNA from brain, testis, liver, kidney, heart, lung, thymus, spleen and cerebellum tissues (RNAs obtained from Clontech). PCR primers were designed to amplify the highly conserved region within the last long coding exon. The UBE1 gene was used as control. (PDF 1306 kb)
Additional file 9: Tests of SPATA31 antibody specificity. A) Test for antibody specificity on bacterially expressed SPATA31 protein. The C-terminal peptide of SPATA31 (starting at aa position 1122, 3362 bp from the M/ATG start codon) was cloned in reverse and forward orientation into the PGEX-4 T1 bacterial expression vector (see scheme) and transformed into Bl21 *E.coli* cells for expression analysis. Coomassie blue staining (above) and Western blot analysis (below) of IPTG induced (1 mM and 0.1 mM for 3 h) cells are shown. A strong antibody signal occurred only for the forward orientation under strong induction. B) Western blot of comparative analysis of SPATA31 protein, stained with affinity purified polyclonal SPATA31 antibody (1:500), from human (HFF cells), macaque cells (CV1) and mouse cells (L929). Cells were fractionated by Qproteom (Qiagen) subcellular fractionation kit, loaded on a 4–15% gradient PAGE-SDS gel (Biorad) and electro-blotted onto a PVDF membrane. Note that the picture shows the cytoplasmic fraction, since this yielded a stronger signal. Mouse monoclonal α-tubulin antibody (Sigma 1:1000) was used as loading control. The predicted gene lengths of the protein coding regions of *SPATA31* genes were 157kD for the A type (A1) and 130kD for the C type (C1) respectively. The detected size on the western blots shows protein bands around 170–200 kD, probably due to post-translational modifications. C) CV1 cells were transiently transfected with C-terminally Flag-tagged *SPATA31A1* (exon4 only, lacking a nuclear localization signal). 24 h after transfection, cells were fixed with cold (4 °C) 1.5% paraformaldehyde at room temperature for 10 min, followed by fixation with Methanol−20 °C for 10 min in−20 °C and stained with DAPI (blue), a monoclonal antibody against Flag-tag (anti-flag-M2 (Sigma, 1:500) (green) and the polyclonal antibody against SPATA31 protein (red). The polyclonal antibody stains both, the protein from cytoplasm expressed Exon4 construct, as well as the endogenous nuclear SPATA31 protein. The scheme of the constructed mammalian expression vector map (pFlag5.1 (Sigma)) is presented below. The figures are merged and magnification is 63X. (PDF 2649 kb)
Additional file 10: Localization of SPATA31 protein in spindle assembly during mitosis. Antibody staining of human primary fibroblast (HFF) cells (63x). Nuclei in the first panel are stained with DAPI (blue), SPATA31 staining is shown in the second panel (red). In the third panel, the green staining in the 63x pictures is cellular cytoskeleton detected with an antibody against tubulin and as a marker for spindle assembly. (PDF 3209 kb)
Additional file 11: Expression and localization of SPATA31 protein in human tissues. Immunohistochemistry staining of SPATA31 protein in fresh frozen tissue sections of Lung CF (A), Sinus (B) and Tonsil allergic (C) (obtained from Swiss Institute of Allergy and Asthma Research, Davos, (SIAF)). Pre-immune_SPATA31, Pre_DAPI (pre-immune and DAPI merged), SPATA31 (1^st^ bleed whole serum, 1:500), SPATA31/DAPI (SPATA31 antibody and DAPI merged). DAPI was used to visualize the nuclei. (PDF 20132 kb)
Additional file 12: Quantification of SPATA31 re-localization within the nucleus upon UVC treatment of HFF cells. The figure depicts % of co-localization between SPATA31 and PER2 antibody within the nucleus upon stimulation with different UVC exposure times (horizontal). Cells were counted from a window of 63X randomly chosen confocal images, containing approximately 20 cells, assessed whether co-localization of SPATA31 in the nucleolus with PER2 occurs upon different UVC treatments. Cells were synchronized for 3 days by replacing IMDM growth medium (including 10% FBS) with serum free IMDM growth media. After 3 days of incubation, serum free growth medium was replaced with IMDM growth medium including 10% FBS. Six hours after addition of serum containing growth media, the growth medium was completely removed from the plates and the cells were irradiated with 0 (control), 10, 20, 40, 80 and 200 J/m^2^ UVC (See methods). After the appropriate UVC irradiation, fresh IMDM growth medium containing FBS was added to each plate and cells were kept 1 h of additional incubation in 37 °C including 5% CO_2_. After 1 h of additional incubation IMDM growth media was removed and cells were fixed immediately with−20 °C precooled 100% methanol for 10 min in a−20 °C freezer in dark conditions. Methanol was then removed and the cells were subjected to immunofluorescence analysis. All UV treatments showed a reduced number of cells with co-localization of SPATA31 in nucleolus with PER2 in different percentages. All differences to control were significant (*P* < 0.01, unpaired two-tailed *t* test). (PDF 49 kb)
Additional file 13: Experimental design for CRISPR/Cas targeted disruption *SPATA31* genes in human. Scheme for the design of the targeted disruption of the open reading frames of *SAPATA31* type A and type C genes (including alternative exons) by using CRISPR/Cas mediated mutagenesis [27]. Six single cell clones were isolated and identified to have different numbers (3-low (S5, S7, S12) and 3-high (S6, S8, S11)) of frame shift mutations. See Additional File 14 for details of this analysis. (PDF 4556 kb)
Additional file 14: Frame Shift Mutation analysis. The upper table provides the number of reads with frameshifts per number of reads for the different annotated *SPATA31* genes, the lower table provides the respective percentages. (PDF 44 kb)
Additional file 15: List of CNVs of *SPATA31* genes in individuals from Chinese, Finnish, Yoruba and Kenyan populations (PDF 88 kb)
Additional file 16: PCR conditions and Primer List. PCR was performed in 20 μL reactions composed of 0.8 μL of a 10 mM dilution of the forward primer and reverse primer, 10 μL of Roche (11636103001) PCR Master Mix. The following PCR conditions were used (A): 3 min at 95 °C, followed by 40 cycles at 95 °C for 30 s, 55 °C 30 s, and 72 °C for 30 s followed by 7 min at 72 °C. The following real-time PCR conditions (B) were used: 3 min at 95 °C, followed by 50 cycles at 95 °C for 15 s, 55 °C 20 s, and 72 °C for 20 s elongation cycle. (PDF 93 kb)

## Abbreviations

CNV: Copy Number Variation
EBV: Epstein Barr Virus
EGF: Epidermal Growth Factor
HFF: Human Foreskin Fibroblast
KLH: Keyhole Limpet Hemocyanin
LDH: Lactate Dehydrogenase
TLS: Trans-lesion Synthesis
UV: Ultraviolet
