# Supplementary figures and images for: Segmental duplications and evolutionary acquisition of UV damage response in the SPATA31 gene family of primates and humans
Source: BMC Genomics. 2017 Mar 6;18:222. doi: 10.1186/s12864-017-3595-8 (PMC5338094; doi:10.1186/s12864-017-3595-8)

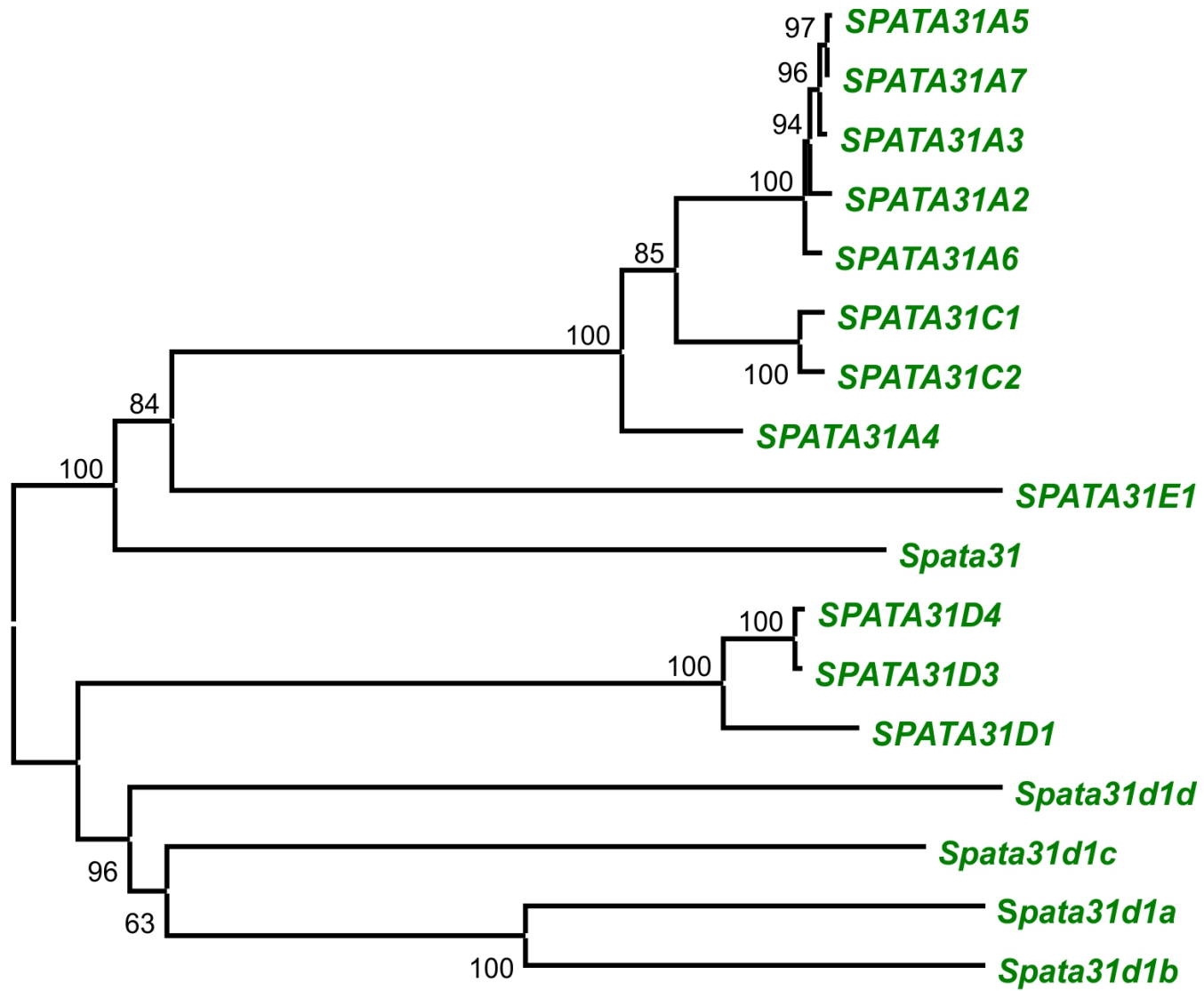

0.05

Supplement: Additional file 1: — Phylogeny of SPATA31 proteins. Neighbor-Joining tree [43] of SPATA31 protein sequences from human (capital letters) and mouse (small letters); bootstrap values (1000 replicates) are shown next to the branches [44]. The tree includes the distant gene families D and E. (PDF 362 kb) [file 12864_2017_3595_MOESM1_ESM.pdf]

Copy Number Estimation

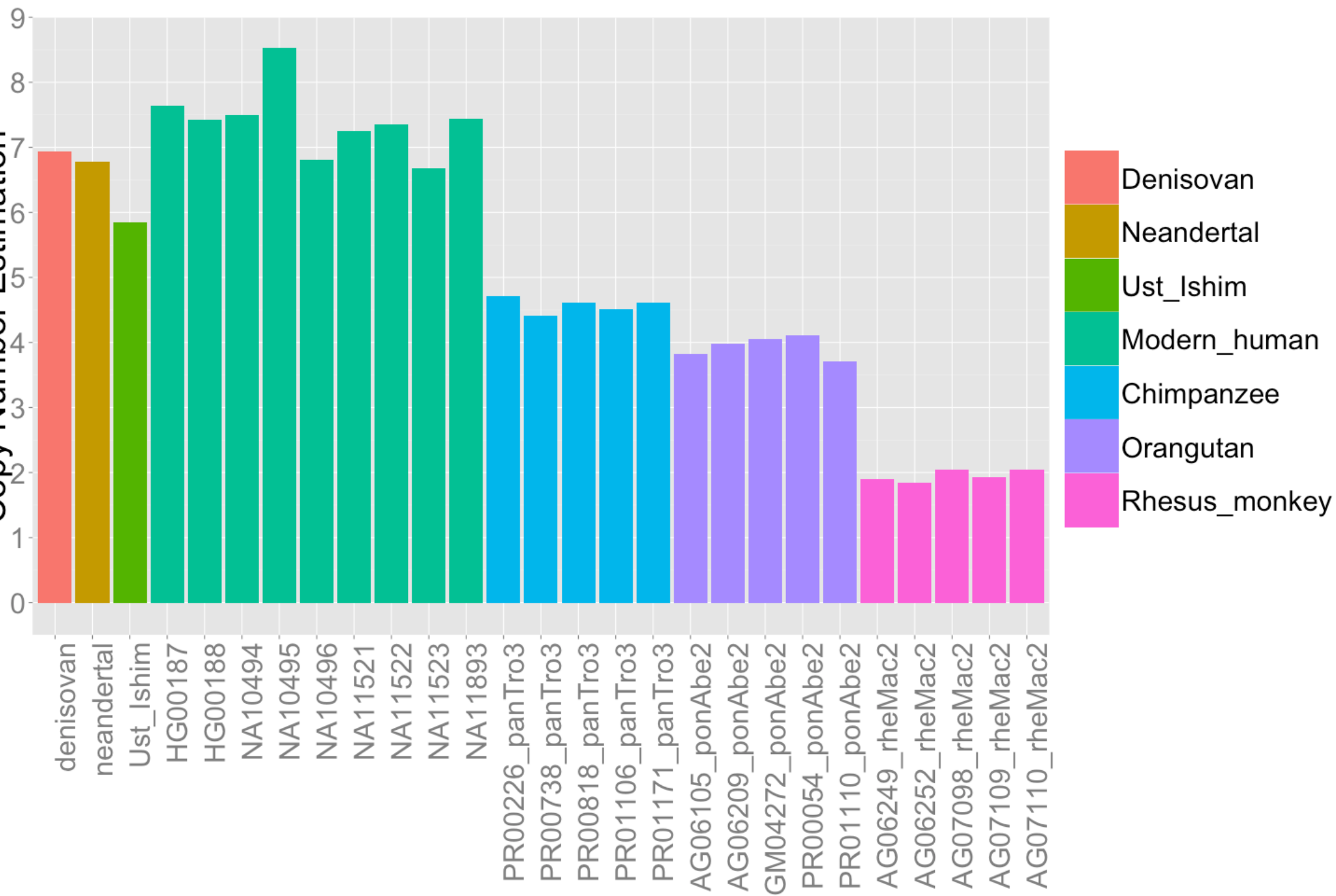

Supplement: Additional file 2: — Copy number estimates of SPATA31 in different lineages of primates. For each genome, the read depth at all putative SPATA31 sites were recorded from the primary genome sequence reads, summed up, and normalized by genome-wide read depth to obtain the haploid copy number estimation (y-axis). The genome samples analyzed are listed on the x-axis, including a Denisovan, a Neandertal, a Ust_Ishim, nine modern humans, five chimpanzees, five orangutans, five rhesus monkeys. Samples are color coded according to their species. Individual ancient genomes were also color-coded. The color-coding is shown on the right of the plot. See Methods for the details of the SPATA31 copy number estimates in primates. (PDF 150 kb) [file 12864_2017_3595_MOESM2_ESM.pdf]

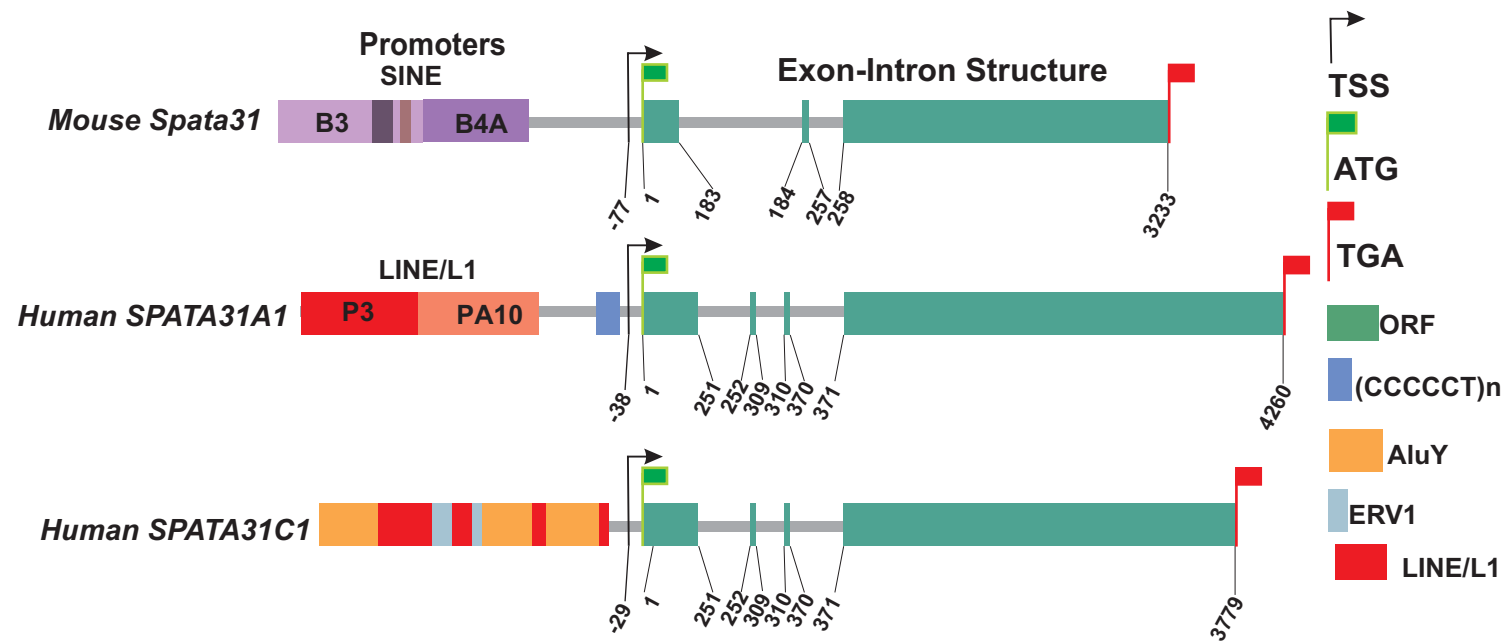

Supplement: Additional file 3: — Promoter comparisons between mouse Spata31 and human SPATA31A/C genes. Overall schematic comparison with the experimentally determined transcriptional start sites indicated with respect to the ATG start codon. To identify the transcription initiation sites, we performed 5’RACE (rapid amplification of cDNA ends). This yielded an initiation site 38 bp upstream of the start codon for SPATA31A, which is consistent with the canonical start site present in sequence databases (note: it differs by the addition of only 18 additional bp when compared to the reference mRNA sequence (NM_001085452) for SPATA31A1. Compared to mouse, we found that LINE/L1 and other elements inserted into the promoter region of SPATA31 genes, creating different upstream regions for A and C types. Further, the type A genes acquired a (CCCCCT)n simple repeat which provides potential Sp1 binding sites, present within the promoter region, which can be traced to the progenitor of humans, gorillas and chimpanzees (compare Fig. 2). (PDF 393 kb) [file 12864_2017_3595_MOESM3_ESM.pdf]

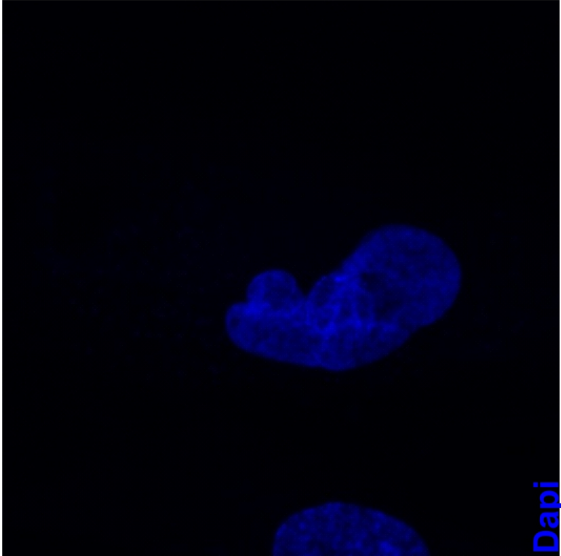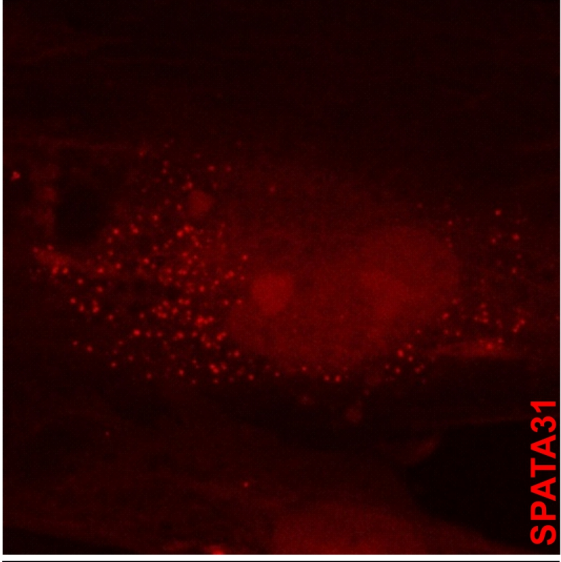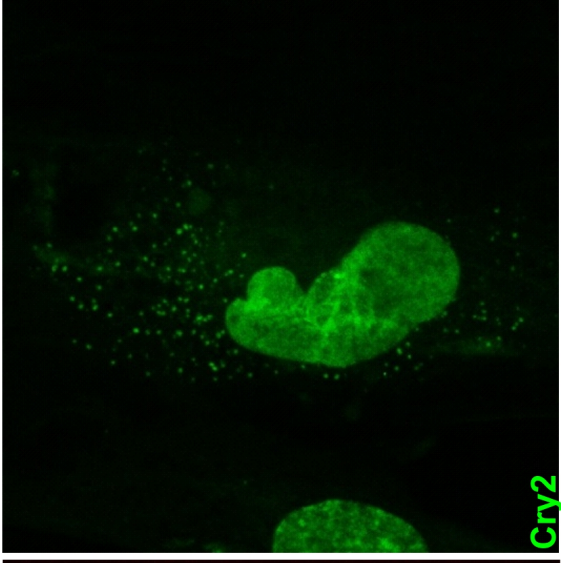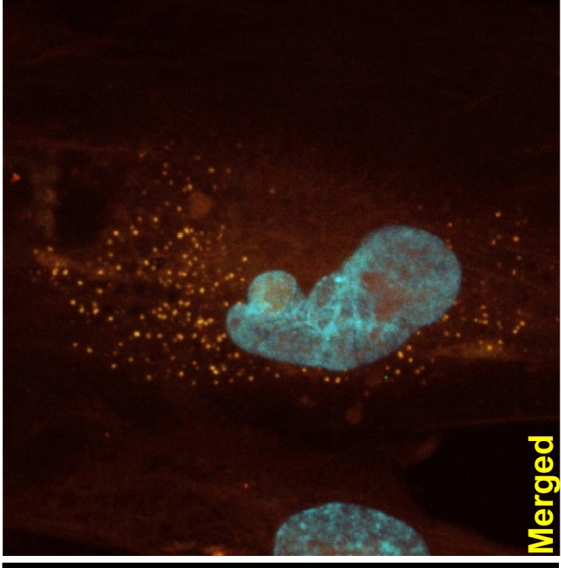

Supplement: Additional file 7: — Co-localization of SPATA31 with CRY2 protein in HFF cell line. Immunofluorescence analysis of SPATA31 protein in HFF cells showing the co-localization of the SPATA31 protein with CRY2 protein. Cells were stained with a 1:100 dilution of SPATA31 antibody (red) and CRY2 antibody (Abcam, 1;250) (green) and merged (yellow). The nucleus is visualized by DAPI staining (blue). Note that these cells had been exposed to light and not syncronized, i.e. the nucleolar staining is not so prominent. (PDF 1403 kb) [file 12864_2017_3595_MOESM7_ESM.pdf]

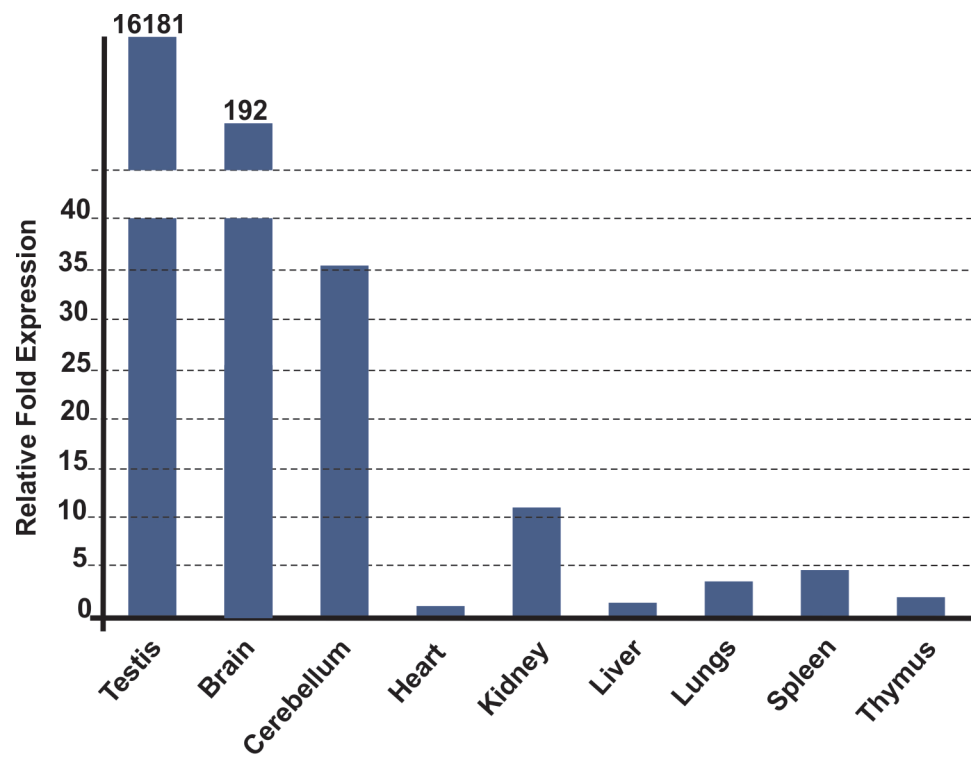

Supplement: Additional file 8: — Quantitative expression analysis of the SPATA31 gene family in human. Quantitative real time PCR analysis on Human SPATA31 genes based on cDNA from brain, testis, liver, kidney, heart, lung, thymus, spleen and cerebellum tissues (RNAs obtained from Clontech). PCR primers were designed to amplify the highly conserved region within the last long coding exon. The UBE1 gene was used as control. (PDF 1306 kb) [file 12864_2017_3595_MOESM8_ESM.pdf]

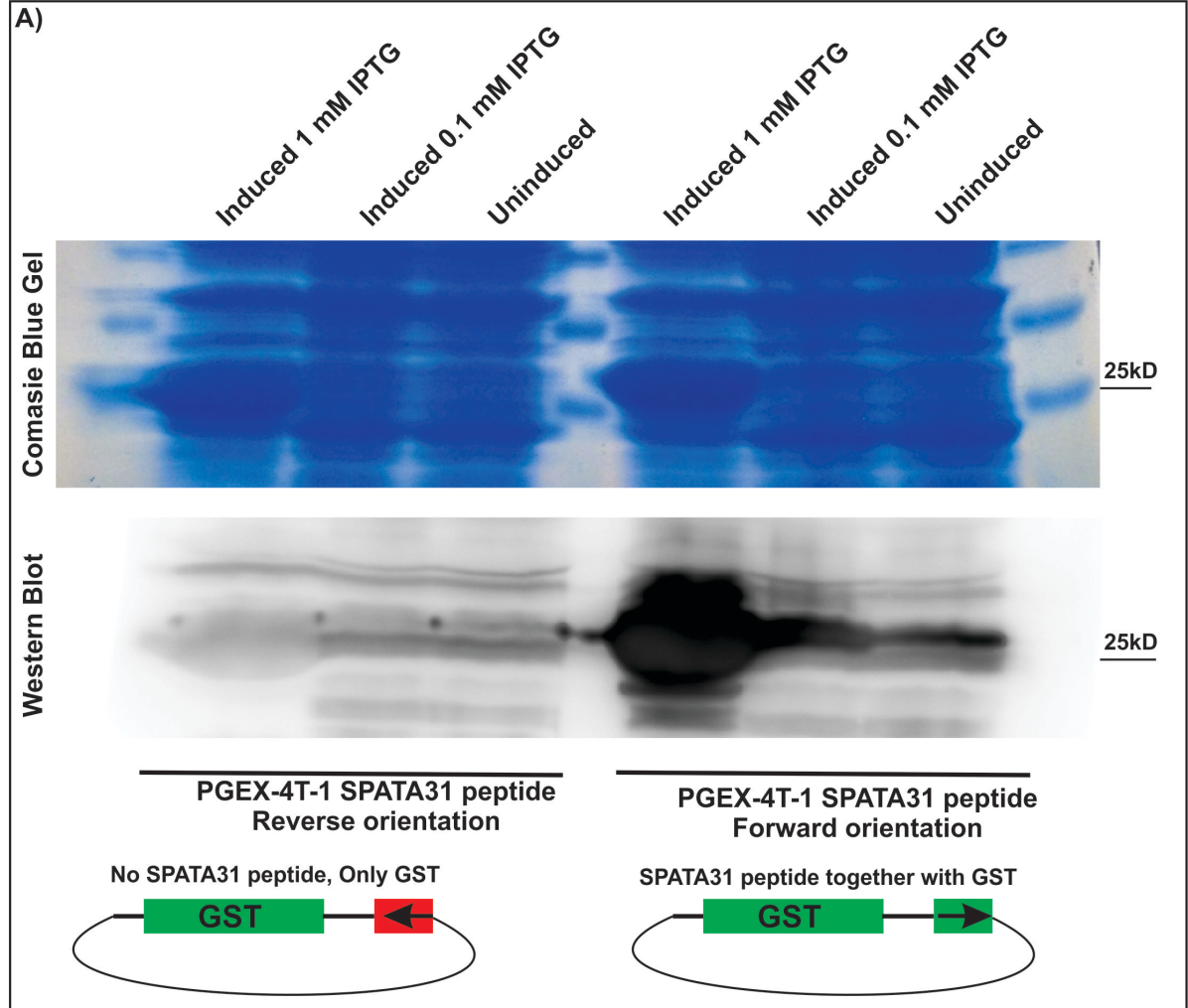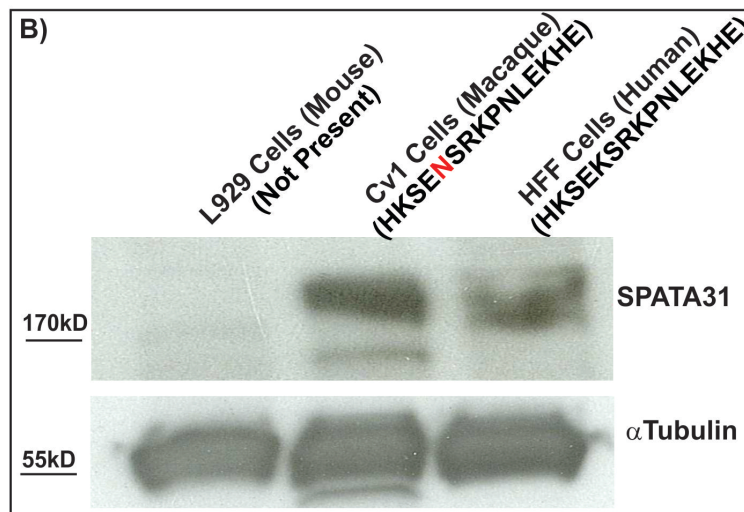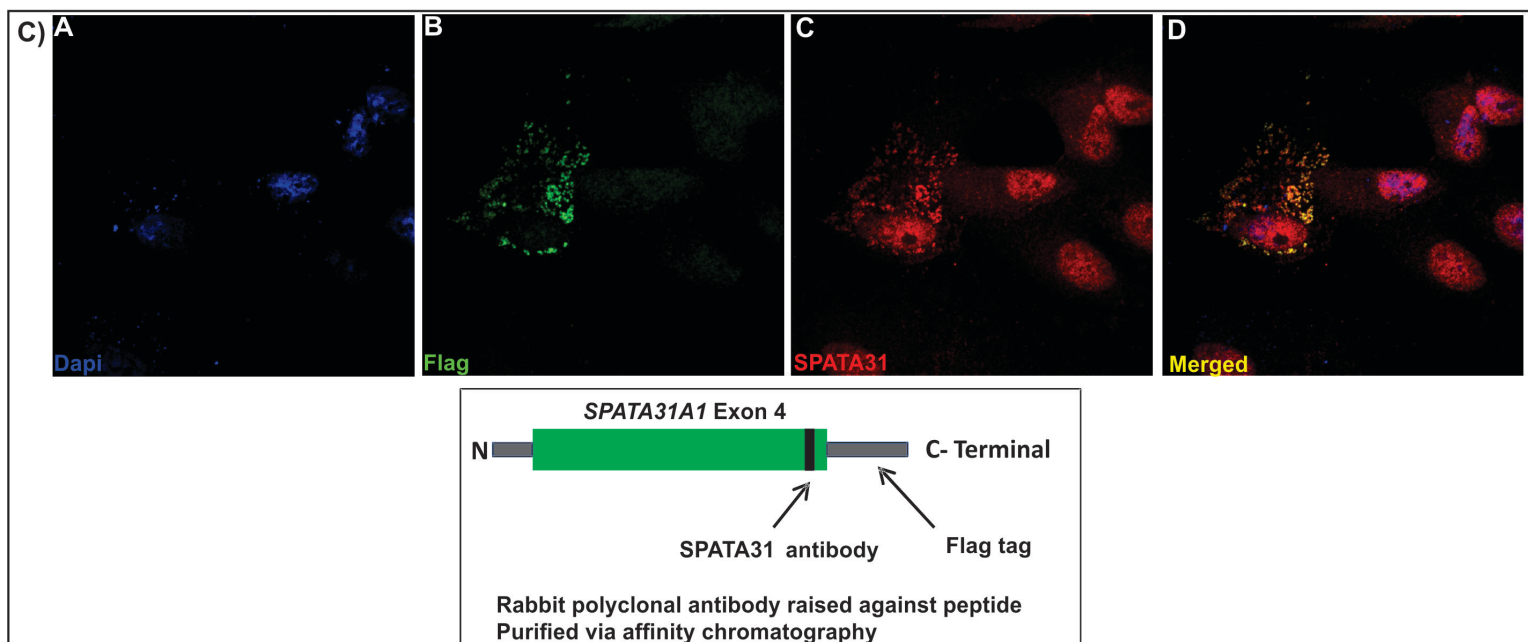

Supplement: Additional file 9: — Tests of SPATA31 antibody specificity. A) Test for antibody specificity on bacterially expressed SPATA31 protein. The C-terminal peptide of SPATA31 (starting at aa position 1122, 3362 bp from the M/ATG start codon) was cloned in reverse and forward orientation into the PGEX-4 T1 bacterial expression vector (see scheme) and transformed into Bl21 E.coli cells for expression analysis. Coomassie blue staining (above) and Western blot analysis (below) of IPTG induced (1 mM and 0.1 mM for 3 h) cells are shown. A strong antibody signal occurred only for the forward orientation under strong induction. B) Western blot of comparative analysis of SPATA31 protein, stained with affinity purified polyclonal SPATA31 antibody (1:500), from human (HFF cells), macaque cells (CV1) and mouse cells (L929). Cells were fractionated by Qproteom (Qiagen) subcellular fractionation kit, loaded on a 4–15% gradient PAGE-SDS gel (Biorad) and electro-blotted onto a PVDF membrane. Note that the picture shows the cytoplasmic fraction, since this yielded a stronger signal. Mouse monoclonal α-tubulin antibody (Sigma 1:1000) was used as loading control. The predicted gene lengths of the protein coding regions of SPATA31 genes were 157kD for the A type (A1) and 130kD for the C type (C1) respectively. The detected size on the western blots shows protein bands around 170–200 kD, probably due to post-translational modifications. C) CV1 cells were transiently transfected with C-terminally Flag-tagged SPATA31A1 (exon4 only, lacking a nuclear localization signal). 24 h after transfection, cells were fixed with cold (4 °C) 1.5% paraformaldehyde at room temperature for 10 min, followed by fixation with Methanol−20 °C for 10 min in−20 °C and stained with DAPI (blue), a monoclonal antibody against Flag-tag (anti-flag-M2 (Sigma, 1:500) (green) and the polyclonal antibody against SPATA31 protein (red). The polyclonal antibody stains both, the protein from cytoplasm expressed Exon4 construct, as well as the endogen [file 12864_2017_3595_MOESM9_ESM.pdf]

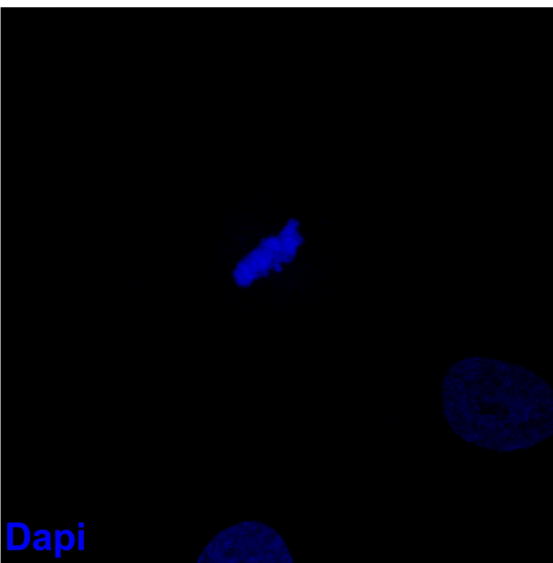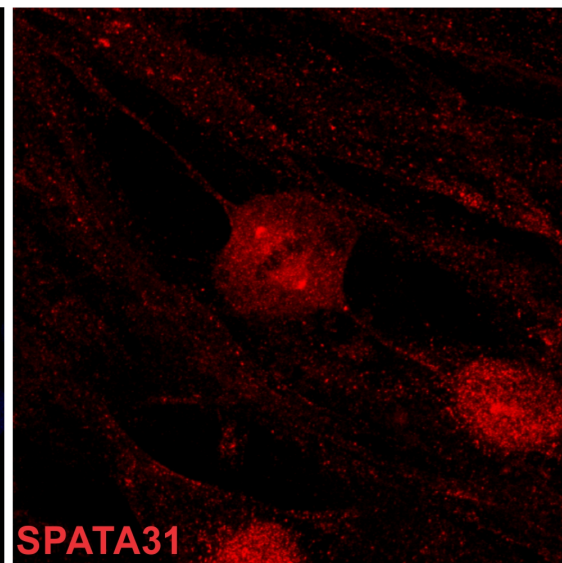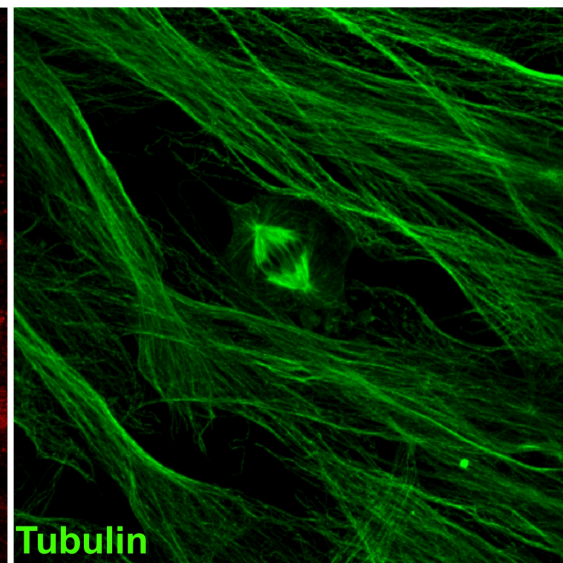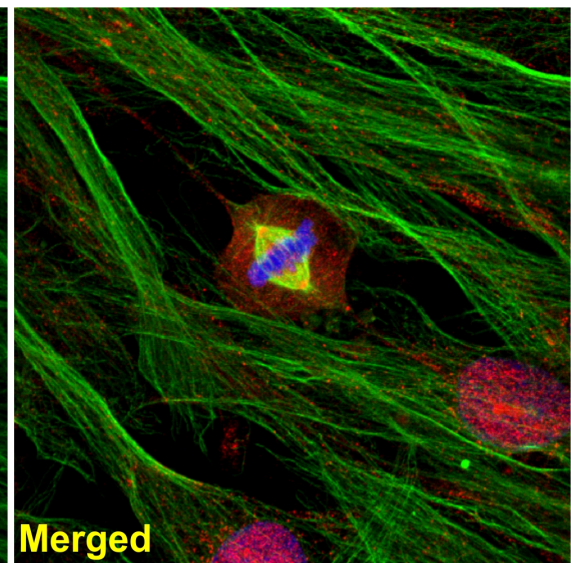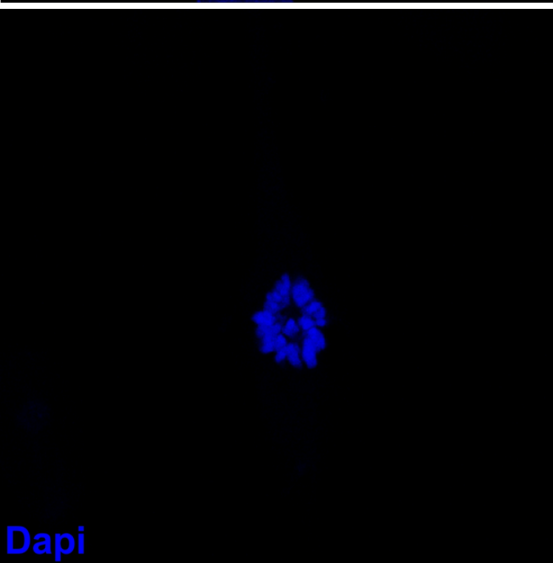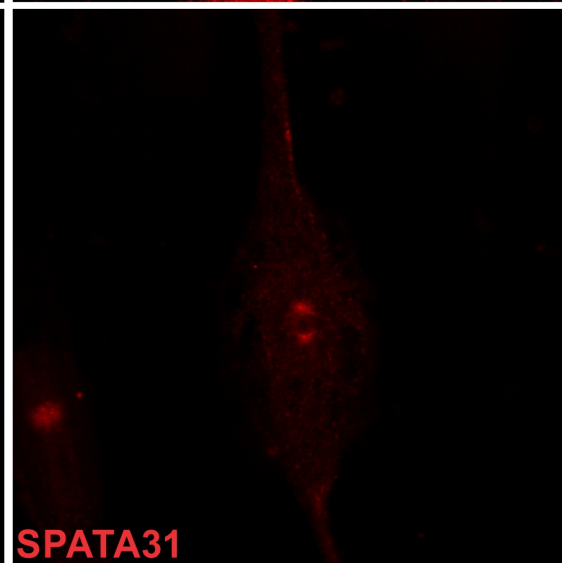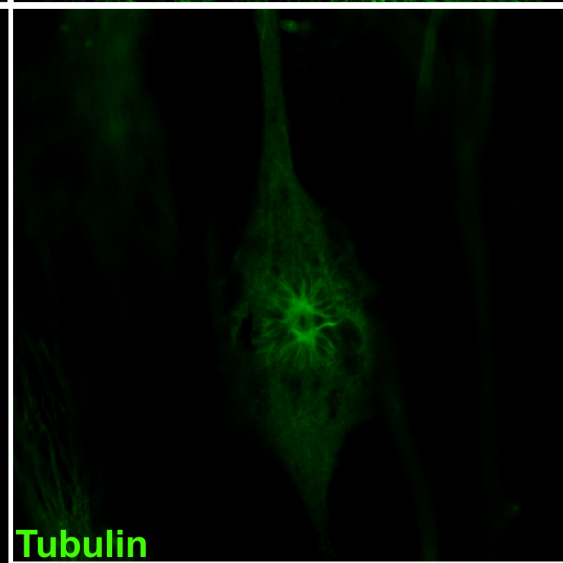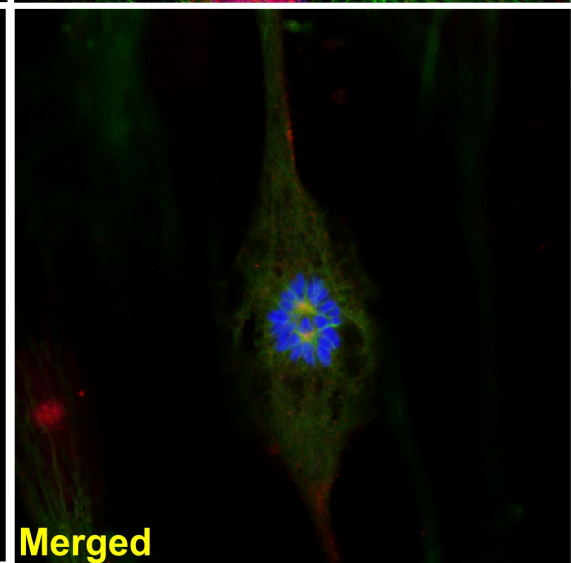

Supplement: Additional file 10: — Localization of SPATA31 protein in spindle assembly during mitosis. Antibody staining of human primary fibroblast (HFF) cells (63x). Nuclei in the first panel are stained with DAPI (blue), SPATA31 staining is shown in the second panel (red). In the third panel, the green staining in the 63x pictures is cellular cytoskeleton detected with an antibody against tubulin and as a marker for spindle assembly. (PDF 3209 kb) [file 12864_2017_3595_MOESM10_ESM.pdf]

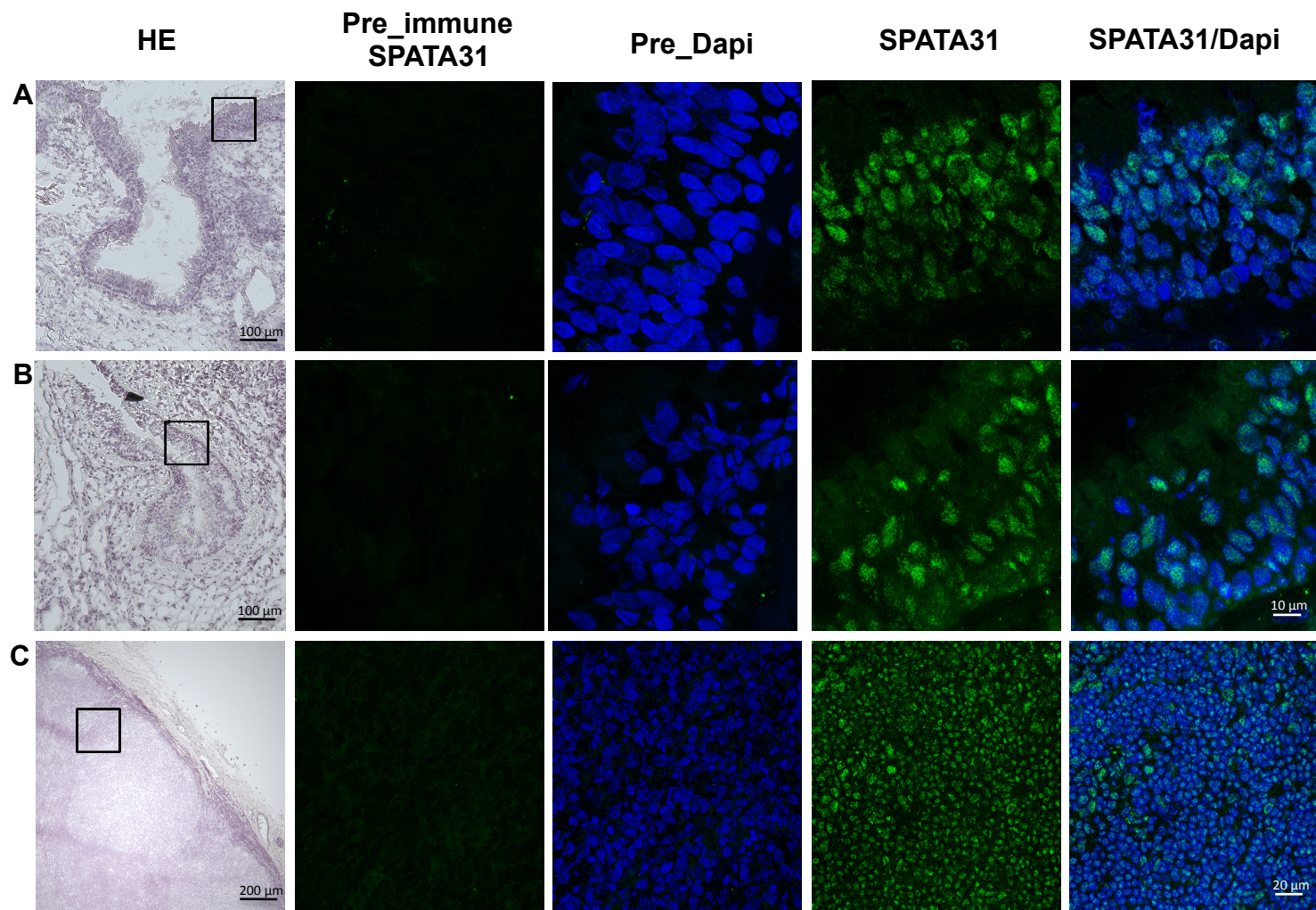

Supplement: Additional file 11: — Expression and localization of SPATA31 protein in human tissues. Immunohistochemistry staining of SPATA31 protein in fresh frozen tissue sections of Lung CF (A), Sinus (B) and Tonsil allergic (C) (obtained from Swiss Institute of Allergy and Asthma Research, Davos, (SIAF)). Pre-immune_SPATA31, Pre_DAPI (pre-immune and DAPI merged), SPATA31 (1st bleed whole serum, 1:500), SPATA31/DAPI (SPATA31 antibody and DAPI merged). DAPI was used to visualize the nuclei. (PDF 20132 kb) [file 12864_2017_3595_MOESM11_ESM.pdf]

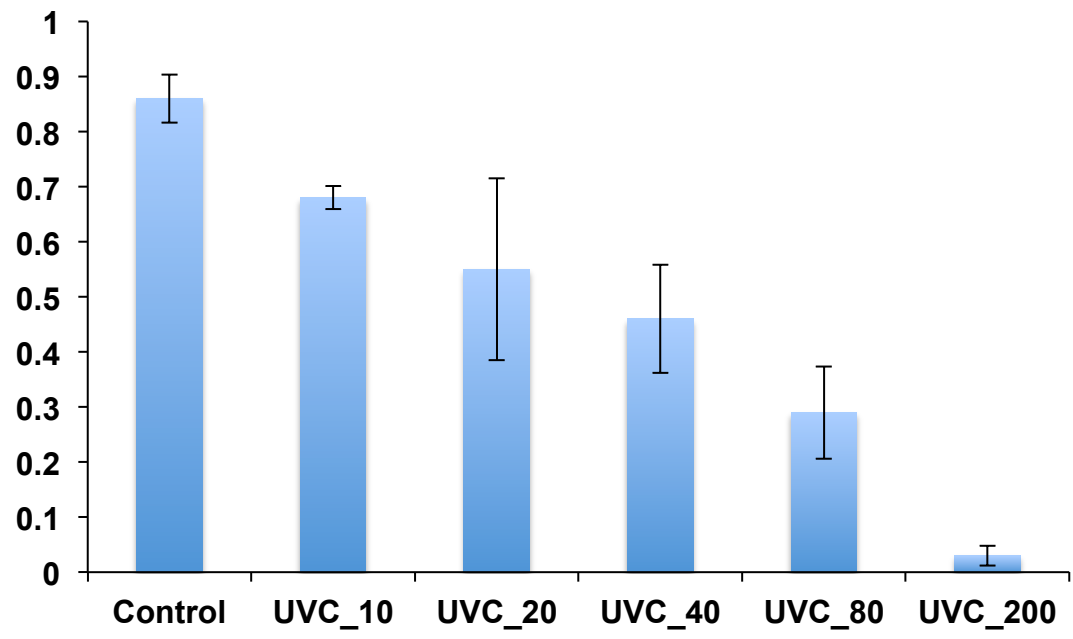

Supplement: Additional file 12: — Quantification of SPATA31 re-localization within the nucleus upon UVC treatment of HFF cells. The figure depicts % of co-localization between SPATA31 and PER2 antibody within the nucleus upon stimulation with different UVC exposure times (horizontal). Cells were counted from a window of 63X randomly chosen confocal images, containing approximately 20 cells, assessed whether co-localization of SPATA31 in the nucleolus with PER2 occurs upon different UVC treatments. Cells were synchronized for 3 days by replacing IMDM growth medium (including 10% FBS) with serum free IMDM growth media. After 3 days of incubation, serum free growth medium was replaced with IMDM growth medium including 10% FBS. Six hours after addition of serum containing growth media, the growth medium was completely removed from the plates and the cells were irradiated with 0 (control), 10, 20, 40, 80 and 200 J/m2 UVC (See methods). After the appropriate UVC irradiation, fresh IMDM growth medium containing FBS was added to each plate and cells were kept 1 h of additional incubation in 37 °C including 5% CO2. After 1 h of additional incubation IMDM growth media was removed and cells were fixed immediately with−20 °C precooled 100% methanol for 10 min in a−20 °C freezer in dark conditions. Methanol was then removed and the cells were subjected to immunofluorescence analysis. All UV treatments showed a reduced number of cells with co-localization of SPATA31 in nucleolus with PER2 in different percentages. All differences to control were significant (P < 0.01, unpaired two-tailed t test). (PDF 49 kb) [file 12864_2017_3595_MOESM12_ESM.pdf]

# CRISPR/Cas9 Fmex1 targeting exon1 of all *SPATA31* genes

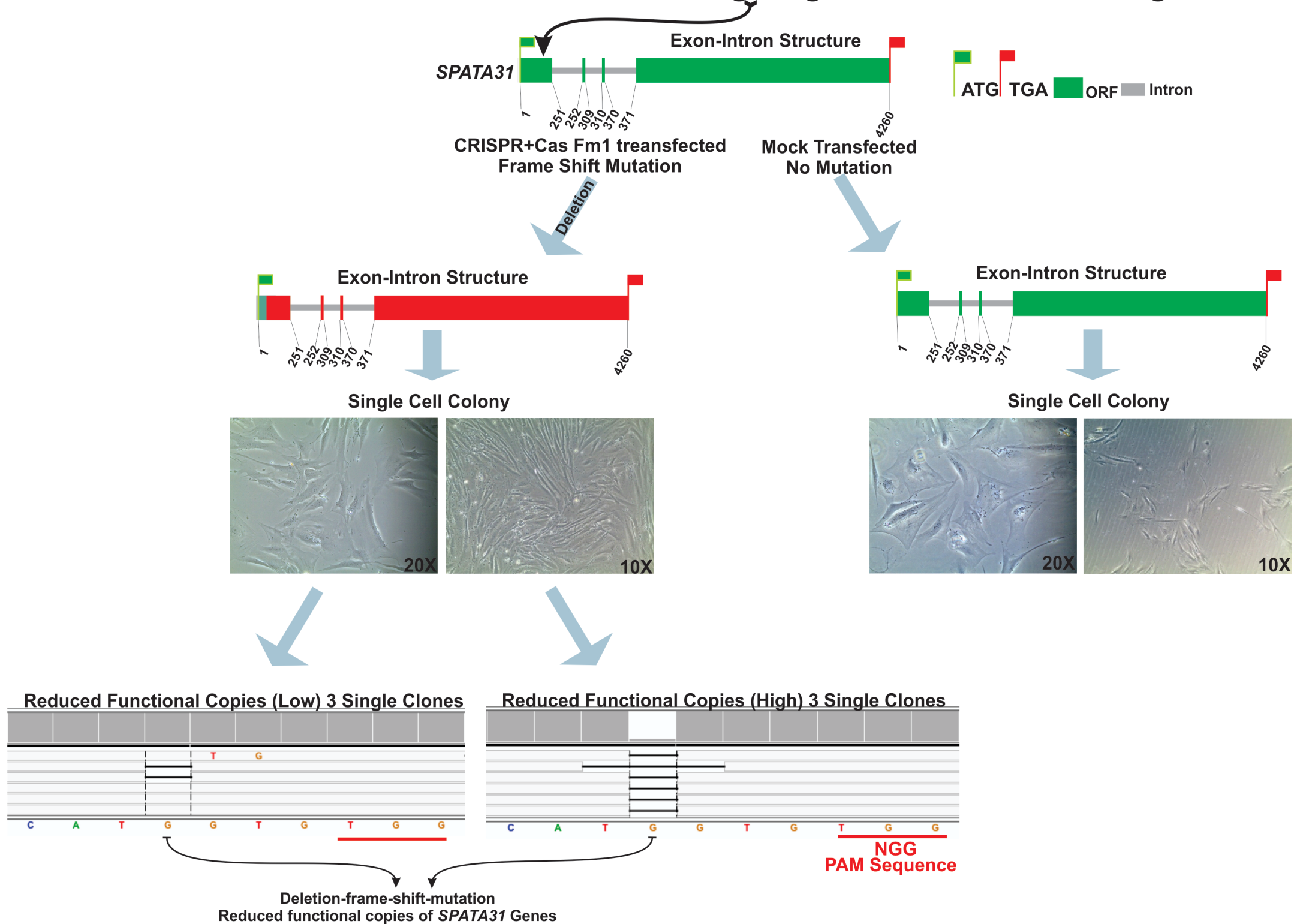

Supplement: Additional file 13: — Experimental design for CRISPR/Cas targeted disruption SPATA31 genes in human. Scheme for the design of the targeted disruption of the open reading frames of SAPATA31 type A and type C genes (including alternative exons) by using CRISPR/Cas mediated mutagenesis [27]. Six single cell clones were isolated and identified to have different numbers (3-low (S5, S7, S12) and 3-high (S6, S8, S11)) of frame shift mutations. See Additional File 14 for details of this analysis. (PDF 4556 kb) [file 12864_2017_3595_MOESM13_ESM.pdf]
