# Supplementary material for: Segmental duplications and evolutionary acquisition of UV damage response in the SPATA31 gene family of primates and humans
Source: BMC Genomics. 2017 Mar 6;18:222. doi: 10.1186/s12864-017-3595-8 (PMC5338094; doi:10.1186/s12864-017-3595-8)
Supplement: Additional file 4: — Alignment of SPATA31 proteins. Alignment of mouse and human SPATA31A/C proteins by Clustal-W [31] and [32]. BoxShade server (http://www.ch.embnet.org/software/BOX_form.html) was used to highlight similar (grey) and identical (black) amino acids. Identified domains corresponding to the ones shown in Fig. 3 are indicated. (PDF 126 kb) [file 12864_2017_3595_MOESM4_ESM.pdf]

```
SPATA31C1    1 MENLPFPLKLLSASSLNTPSSTPWVLDIFLTLVFALGLFFLLLPYFSYLRCDNPPSPSPRKRKRHLVSQRHLVSQCPTGR
SPATA31C2    1 MENLPFPLKLLSASSLNTPSSTPWVLDIFLTLVFALGFFFLLLPYFSYLRCDNPPSPSPKKRKRHLVSQR------PAGR
SPATA31A4    1 MENLPFPLKLLSASSLNTPSSTPWVLDIFLTLVFALGFFFLLLPYFSYLRCDDPPSPSPGKRK------------CPVGQ
SPATA31A5    1 MENLPFPLKLLSASSLNAPSSTPWVLDIFLTLVFALGFFFLLLPYLSYFRCDDPPSPSPGKRK------------CPVGR
SPATA31A7    1 MENLPFPLKLLSASSLNAPSSTPWVLDIFLTLVFALGFFFLLLPYLSYFRCDDPPSPSPGKRK------------CPVGR
SPATA31A3    1 MENLPFPLKLLSASSLNAPSSTPWVLDIFLTLVFALGFFFLLLPYLSYFRCDDPPSPSPGKRK------------CPVGR
SPata31A2    1 MENLPFPLKLLSASSLNAPSSTPWVLDIFLTLVFALGFFFLLLPYLSYFRCDDPPSPSPGKRK------------CPVGR
SPATA31A6    1 MENLPFPLKLLSASSLNAPSSTPWVLDIFLTLVFALGFFFLLLPYLSYFHCDDPPSPSPGKRK------------CPVGR
spata31      1 MESLPSLLESIYTTWLSLSS-TIWAMDMTLAFVCGLGLYHLLLPFLESHLSSPPSNIKFTRKPQ-----------IQMTW
                              Transmembrane Domain                          Nuclear Localization Signal

SPATA31C1   81 RGRPRGRMKNHS---LRACRECPRGLEETWDLLSQLQSLLGPHLEKGDFGQLSGPDPPGEVGKRTPDGASRSSHEPMEDA
SPATA31C2   75 RGRPRGRMKNHS---LRACRECPRGLEETWDLLSQLQSLLGPHLEKGDFGQLSGPDPPGEVGKRTPDGASRSSHEPTEDA
SPATA31A4   69 RGRPRGRMKNHS---LRACRECPRGLEETWDLLSQLQSLLGTHLDKGDFGQLSGPEPPGEVGKRAPDGASRSSLEPMEDA
SPATA31A5   69 RRRPRGRMKNHS---LRAGRECRRGLEETSDLLSQLQSLLGPHLDKGDFGQLSGPDPPGEVGERAPDGASQSSHEPMEDA
SPATA31A7   69 RRRPRGRMKNHS---LRAGRECPRGLEETSDLLSQLQSLLGPHLDKGDFGQLSGPDPPGEVGERAPDGASQSSHEPMEDA
SPATA31A3   69 RRRPRGRMKNHS---LRAGRECRRGLEETSDLLSQLQSLLGPHLDKGDFGQLSGPDPPGEVGERAPDGASQSSHEPMEDA
SPata31A2   69 RRRPRGRMKNHS---LRAGRECRRGLQETSDLLSQLQSLLGPHLDKGDFGQLSGPDPPGEVGERAPDGASQSSHEPMEDA
SPATA31A6   69 RRRPRGRMKNHS---LRAGRECPRGLEETSDLLSQLQSLLGPHLDKGDFGQLSGPDPPGEVGERAPDGASQSSHEPMEDA
spata31     69 QSQFKKKFRNHCRNDAKAWGECLKKLKEKEKDKLFLEEMSPGHHLNSLGNIFNSSSAKQDSTTLSPFWNLKEKSEEQVAT
               Nuclear Localization Signal

SPATA31C1  158 APIVSPLASPDPRTKHP----QDLASTPPPGPMTTSVSSLSASQPPEPSLLLERPSPEPPALFPHPPHTPDPLACSPPPP
SPATA31C2  152 APIVSPLASPDPRTKHP----QDLASTPPPGPMTTSVSSLSASQPPEPSLLLEHPSPEPPALFPHPPRTPDPLACSPPPP
SPATA31A4  146 APIIYLLASPDPRIKHP----QDLASTPSPGPMTTSVSSLSASQPPEPSLLLERPSPKPPALF----------------
SPATA31A5  146 APILSPLASPDPQAKHP----QDLASTPSPGPMTTSVSSLSASQPPEPSLPLEHPSPEPPALFPHPPHTPDPLACSLPPP
SPATA31A7  146 APILSPLASPDPQAKHP----QDLASTPSPGPMTTSVSSLSASQPPEPSLPLEHPSPEPPALFPHPPHTPDPLACSLPPP
SPATA31A3  146 APILSLLASPDPQAKHP----QDLASTPSPGPMTTSVSSLSASQPPEPSLPLEHPSPEPPALFPHPPHTPDPLACSLPPP
SPata31A2  146 APILSPLASPDPQAKHP----QDLASTPSPGPMTTSVSSLSASQPPEPSLPLEHPSPEPPALFPHPPHTPDPLACSPPPP
SPATA31A6  146 APILSPLASPDPQAKHP----QDLASTPSPGPMTTSVSSLSASQPPEPSLPLEHPSPEPPALFPHPPHTPDPLACSPPPP
spata31    149 QKLSYPKISEDHFQQKCDQLFWGLPSLHSESLVAAAWIPQTTSTLPSPFFLFNVISSVYPIQLQD---------------
                                                                 Cryptochrome/photolyase Domain
                                       Cry2 similar region

SPATA31C1  234 KGFTPPPLRDSTLLTPSHCDSVALPLDTVPQSLSPREDLAASVPAISGLGGSNSQVSALSWSQETTKTWCIFNSSVQQDH
SPATA31C2  228 KGFTPPPLRDSTLLTPSHCDSVALPLDTVPQSLSPREDLAASVPGISGLGGSNSQVSALSWSQETTKTWCVFNSSVQQDH
SPATA31A4  205 --------------------------------------------------------------------------------
SPATA31A5  222 KGFTAPPLRDSTLITPSHCDSVAFPLGTVPQSLSPHEDLVASVPAISGLGGSNSHVSASSRWQETARTSCAFNSSVQQDH
SPATA31A7  222 KGFTAPPLRDSTLITPSHCDSVAFPLGTVPQSLSPHEDLVASVPAISGLGGSNSHVSASSRWQETARTSCAFNSSVQQDH
SPATA31A3  222 KGFTAPPLRDSTLITPSHCDSVALPLGTVPQSLSPHEDLVASVPAISGLGGSNSHVSASSRWQETARTSCAFNSSVQQDH
SPata31A2  222 KGFTAPPLRDSTLITPSHCDSVALPLGTVPQSLSPHEDLVASVPAISGLGGSNSHVSASSRWQETARTSCAFNSSVQQDH
SPATA31A6  222 KGFTAPPLRDSTLITPSHCDSVALPLGTVPQSLSPHEDLVASVPAISGLGGSNSHVSASSRWQETARTSCAFNSSVQQDP
spata31    214 --------------------------------------------------------------------------------
               Cryptochrome/photolyase Domain
               Cry2 similar region

SPATA31C1  314 LSRQRD--------------------------------------------------------------------------
SPATA31C2  308 LSRQRD--------------------------------------------------------------------------
SPATA31A4  205 LTHHTP--------------------------------------------------------------------------
SPATA31A5  302 LSRHPPETCQMEAGSLFLLSSDGQNVVGIQVTETAKVNIWEEKENVGSFTNRMTPEKHLNSLRNLAKSLDAEQDTTNPKP
SPATA31A7  302 LSRHPPETCQMEAGSLFLLSSDGQNVVGIQVTETAKVNIWEEKENVGSFTNRMTPEKHLNYLRNLAKSLDAEQDTTNPKP
SPATA31A3  302 LSRHPPETCQMEAGSLFLLSSDGQNVVGIQVTETAKVNIWEEKENVGSFTNRMTPEKHLNSLRNLAKSLDAEQDTTNPKP
SPata31A2  302 LSRHPPETYQMEAGSLFLLSSDGQNAVGIQVTETAKVNIWEEKENVGSFTDRMTPEKHLNSLRNLAKSLDAEQDTTNPKP
SPATA31A6  302 LSRHPPETCQMEAGSLFLLSSDGQNVVGIQVTETAKVNIWEEKENVGSFTNQMTPEKHLNSLGNLAKSLDAEQDTTNPKP
spata31    214 --------------------------------------------------------------------------------

SPATA31C1  320 ------------------------------------------------------------------------------TT
SPATA31C2  314 ------------------------------------------------------------------------------TT
SPATA31A4  211 ------------------------------------------------------------------------------LI
SPATA31A5  382 FWNMGENSKQLPGPQKLSDPRLWQESFWKNYSQLFWGLPSLHSESLVANAWVTDRSYTLQSPPFLFNEMSNVCPIQRETT
SPATA31A7  382 FWNMGENSKQLPGPQKLSDPRLWQESFWKNYSQLFWGLPSLHSESLVANAWVTDRSYTLQSPPFLFNEMSNVCPIQRETT
SPATA31A3  382 FWNMGENSKQLPGPQKLSDPRLWQESFWKNYSQLFWGLPSLHSESLVANAWVTDRSYTLQSPPFLFNEMSNVCPIQRETT
SPata31A2  382 FWNMGENSKQLPGPQKFSDPRLWQESFWKNYSQLFWGLPSLHSESLVANAWVTDRSYTLQSPPFLFNEMSNVCPIQRETT
SPATA31A6  382 FWNMGENSKQLPGPQKCSDPRLLQESFWKNYSQLFWGLPSLHSESLVANAWVTDRSYTLQSPPFLFNEMSNVCPIQRETT
spata31    214 -------------------------------------------------------------------------------K
                                    FAM75A Domain

SPATA31C1  322 MSPLLFQAQPLSHLGPESQPFISSTPQFRPTPMAQAEAQAHLQSSFPVLSPAFLSPMKNTGVACPASQNKVQALSLPETQ
SPATA31C2  316 MSPLLFQAQPLSHLEPESQPFISSTPQFWPTPMAQAEAQAHLQSSFPVLSPAFLSPMKNTGVACPASQNKVQALSLPETQ
SPATA31A4  213 LWPALLLLQKTSLLLPCGTPL-----------------------------------------------------------
SPATA31A5  462 MSPLLFQAQPLSHLGPECQPFISSTPQFRPTPMAQAEAQAHLQSSFPVLSPAFPSLIQNTGVACPASQNKVQALSLPETQ
SPATA31A7  462 MSPLLFQAQPLSHLGPECQPFISSTPQFRPTPMAQAEAQAHLQSSFPVLSPAFPSLIQNTGVACPASQNKVQALSLPETQ
SPATA31A3  462 MSPLLFQAQPLSHLGPECQPFISSTPQFRPTPMAQAEAQAHLQSSFPVLSPAFPSLIQNTGVACPASQNKVQALSLPETQ
SPata31A2  462 MSPLLFQAQPPSHLGPECQPFISSTPQFRPTPMAQAEAQAHLQSSFPVLSPAFPSLIKNTGVACPASQNKVQALSLPETQ
SPATA31A6  462 MSPLLFQAQPLSHR----QPFISSTPQFLPTPMAQAEAQAHLQSSFPVLSPAFPSLIKNTGVACPASQNKVQALSLPETQ
spata31    215 MSPMLPHTHPLSYLDLQSPHLILSPLEEQTP-----------ALNLPVLLPSSLPYNSDFGTSYSQSQSKPQYLPT-EIK
               FAM75A Domain

SPATA31C1  402 HPERPLLRKQLEGGLALPSRVQKSQDVFSVSTPNLPQERLTSILPENFPVSPELWRQLEQYM---------GQRGRIQES
SPATA31C2  396 HPERPLLKKQLEGGLALPSRVQKSQDVFSVSTPNLPQERLTSILPENFPVSPELWRQLEQHM---------GQRGRIQES
SPATA31A4      --------------------------------------------------------------------------------
```

```
SPATA31A5   542  HPEWPLLRRQLEGRLALPSRVQKSQDVFSVSTPNLPQESLTSILPENFPVSPELRRQLEQHIKKWIIQH-WGNLGRIQES
SPATA31A7   542  HPEWPLLRRQLEGRLALPSRVQKSQDVFSVSTPNLPQESLTSILPENFPVSPELRRQLEQHIKKWIIQH-WGNLGRIQES
SPATA31A3   542  HPEWPLLRRQLEGRLALPSRVQKSQDVFSVSTPNLPQESLTSILPENFPVSPELRRQLEQHIKKWIIQH-WGNLGRIQES
SPata31A2   542  HPEWPLLRRQLEGRLALPSRVQKSQDVFSVSTPNLPQESLTSILPENFPVSPELRRQLEQHIKKWIIQH-WGNLGRTQES
SPATA31A6   538  HPEWPLLRKQLEGRLALPSRVQKSQDVFSVSTPNLPQESLTSILPENFPVSPELRRQLEQHIKKWIIQH-WGNLGRIQES
spata31     283  YNKKPSLAKQIENRSTLPLMVQKPQEAYDILAPNPSQDWVVSILPDNFPICRELREKLEQHIQKWLIQHQWNFPHKIQVS
                                                 Type 1 DNA Topoisomerase Domain

SPATA31C1   473  LDLMQLQDELPGTSQAKGKPRPWQSSTSTGESSKEAQTVKFQLERDPCPHLGQILGETPQNLSRGMESFPGKVLGATSEE
SPATA31C2   467  LDLMQLQDELPGTSQAKGKPRPWQSSTSTGESSKEAQTVKFQLERDPCPHLGQILGETPQNLSRGMESFPGKVLGATSEE
SPATA31A4        --------------------------------------------------------------------------------
SPATA31A5   621  LDLMQLRDESPGTSQAKGKPSPWQSSMSTGEGSKEAQKVKFQLERDPCPHLGQILGETPQNLSRDMKSFPRKVLGVTSEE
SPATA31A7   621  LDLMQLRDESPGTSQAKGKPSPWQSSMSTGESSKEAQKVKFQLERDPCPHLGQILGETPQNLSRDMKSFPRKVLGVTSEE
SPATA31A3   621  LDLMQLQDESPGTSQAKGKPSPWQSSMSTGESSKEAQKVKFQLERDPCPHLGQILGETPQNLSRDMKSFPRKVLGVTSEE
SPata31A2   621  LDLMQLRDESPGTSQAKGKPSPWQSSMSTGESSKEAQKVKFQLERDPCPHLGQILGETPQNLSRDMKSFPRKVLGVTSEE
SPATA31A6   617  LDLMQLRDESPGTSQAKGKPSPWQSSTSTGESSKEAQKVKFQLERDLCPHLGQILGETPQNLSRDMKSFPRKVLGVTSEE
spata31     363  EKMKELQNTVIGNCQTRDKPG---TLQAIGEHSNEGQKRKFQLEKESGKNLGPILGKISK--------------------
                 FAM75A Domain                                            Nuclear Localization Signal

SPATA31C1   553  SERNLRKPLRSDSGSDLLRRTERNHIENILKAHMGRKLGQTNEGLIPVSVRRSWLAVNQAFPVSNTHVKTSNLAAPKSRK
SPATA31C2   547  SERNLRKPLRSDSGSDLLRRTERNHIENILKAHMSRKLGQTNEGLIPVSVRRSWLAVNQAFPVSNTHVKTSNLAAPKSRK
SPATA31A4        --------------------------------------------------------------------------------
SPATA31A5   701  LERNLRKPLRSDSGSDLLRCTERTHIENILKAHMGRNLGQTNEGLIPVCVRRSWLAVNQALPVSNTHVKTSNLAAPKSGK
SPATA31A7   701  LERNLRKPLRSDSGSDLLRCTERTHIENILKAHMGRNLGQTNEGLIPVCVRRSWLAVNQALPVSNTHVKTSNLAAPKSGK
SPATA31A3   701  LERNLRKPLRSDSGSDLLRCTERTHIENILKAHMGRNLGQTNEGLIPVRVRRSWLAVNQALPVSNTHVKTSNLAAPKSGK
SPata31A2   701  SERNLRKPLRSDSGSDLLRCTERTHIENILKAHMGRNLGQTNEGLIPVRVRRSWLAVNQALPVSNTHVKTSNLAAPKSGK
SPATA31A6   697  SERNLRKPLRSDSGSDLLRCTERTHIENILKAHMGRNLGQTNEGLIPVRVRRSWLAVNQALPVSNTHVKTSNLAAPKSGK
spata31     420  -----------DPIRGLEKTTAIKDLENNLKAHLGTKSGQIDQGLTPLSMRQSWLAVDDSFYEMEN-----NLTSLKSSA
                 FAM75A Domain
                 Nuclear Localization Signal

SPATA31C1   633  ACVNTAQVLSFLELCTQQVLEAHIVRFWAKHRWGLPLRVLKPIQCFQLEKVSSLSLIQLAGPSSDTCESGAGSKVEVATL
SPATA31C2   627  ACVNTAQVLSFLEPCTQQVLGAHIVRFWAKHRWGLPLRVLKPIQCFQLEKVSSLSLIQLAGPSSDTCESGAGSKVEVATF
SPATA31A4        --------------------------------------------------------------------------------
SPATA31A5   781  ACVNTAQVLSFLEPCTQQGLGAHIVRFWAKHRWGLPLRVLKPIQCFKLEKVSSLSLTQLAGPSSATCESGAGSEVEVDMF
SPATA31A7   781  ACVNTAQVLSFLEPCTQQGLGAHIVRFWAKHRWGLPLRVLKPIQCFKLEKVSSLSLTQLAGPSSATCESGAGSEVEVDMF
SPATA31A3   781  ACVNTAQVLSFLEPCTQQGLGAHIVRFWAKHRWGLPLRVLKPIQCFKLEKVSSLSLTQLAGPSSATCESGAGSEVEVDMF
SPata31A2   781  ACVNTAQVLSFLEPCTQQGLGAHIVRFWAKHRWGLPLRVLKPIQCFKLEKVSSLSLTQLAGPSSATCESGAGSEVEVDMF
SPATA31A6   777  ACVNTAQVLSFLEPCTQQGLGAHIVRFWAKHRWGLPLRVLKPIQCFKLEKVSSLSLTQLAGPSSATCESGAGSEVEVDMF
spata31     484  KSMCSSEKLAFLKPETRQALEAHIVRFWAKHRWSLPLKILKPVKLFQLS-IESLPVVLCAQTSSTTSVHRTRSAAEVVRF

SPATA31C1   713  IGEPPMASLRKQVLTKPSVHMPERLQASSPACKQFQRAPRGIPSSNDHGSLKAPTAGQEGRWPSKPLTYSLKGSTQQSRS
SPATA31C2   707  IGEPPMASLRKQVLTKPSVHMPERLQASSPACKQFQRAPRGIPSSNDHGSLKAPTAGQEGRWPSKPLTYSLTGSTQQSRS
SPATA31A4        --------------------------------------------------------------------------------
SPATA31A5   861  LRKPPMASLRKQVLTKASDHMPESLLASSPAWKQFQRAPRGIPSWNDHEPLKPPPAGQEGRWPSKPLTYSLTGSIQQSRS
SPATA31A7   861  LRKPPMASLRKQVLTKASDHMPESLLASSPAWKQFQRAPRGIPSWNDHEPLKPPPAGQEGRWPSKPLTYSLTGSIQQSRS
SPATA31A3   861  LRKPPMASLRKQVLTKASDHMPESLLASSPAWKQFQRAPRGIPSWNDHEPLKPPPAGQEGRWPSKPLTYSLTGSTQQSRS
SPata31A2   861  LRKPPMASLRKQVLTKASDHMPESLLASSPAWKQFQRAPRGIPSWNDHGPLKPPPAGQEGRWPSKPLTYSLTGSTQQSRS
SPATA31A6   857  LRKPPMASLRKQVLTKASDHMPESLLASSPAWKQFQRAPRGIPSWNDHGPLKPPPAGQEGRWPSKPLTYSLTGSTQQSRS
spata31     563  LGKP---CLRQMIIEDSSPSPQNLLLVSSPSCKRAR---RRLPFGVDHEPSTALPTKPECGHISEDLTYNFMNITSQIRT

SPATA31C1   793  LGAQSSRAGETREAVPQPTVPLGTCMRANLQATSEDVRGFKAPGASKSSLLPRMSVSQDPRKLCLMEEAVSEFEPGMATK
SPATA31C2   787  LGAQSSRAGETREAVPQPTVPLGTCMRANLQATSEDVRGFKAPGASKSSLLPRMSVSQDPRKLCLMEEAVSEFEPGKATK
SPATA31A4        --------------------------------------------------------------------------------
SPATA31A5   941  LGAQSSKAGETREAVPQCRVPLETCMLANLQATSEDVHGFEAPGTSKSSLHPRVSVSQDPRKLCLMEEVVNEFEPGMATK
SPATA31A7   941  LGAQSSKAGETREAVPQCRVPLETCMLANLQATSEDVHGFEAPGTSKSSLHPRVSVSQDPRKLCLMEEVVNEFEPGMATK
SPATA31A3   941  LGAQSSKAGETREAVPQCRVPLETCMLANLQATSEDVHGFEAPGTSKSSLHPRVSVSQDPRKLCLMEEVVSEFEPGMATK
SPata31A2   941  LGAQSSKAGETREAVPQCRVPLETCMLANLQATSEDMHGFEAPGTSKSSLHPRVSVSQDPRKLCLMEEVVNEFEPGMATK
SPATA31A6   937  LGAQSSKAGETREAVPQCRVPLETCMLANLQATSEDVHGFEAPGTSKSSLHPRVSVSQDPRKLCLMEEVVSEFEPGMATK
spata31     637  IFKKEIET------------------------------------REVVLLPRRTSDQNLEAYKHQEKVVSEFPHNVETE

SPATA31C1   873  SETQPQVSAAVVLLPDGQASVVPHASENLASQVPQGHLQSTPTGNMQASQELCDLMSARRSNVGHKEPRNPNCQGSCKSQ
SPATA31C2   867  SETQPQVSATVVLLPDGQASVVPHASENLASQVPQGHLQSMPTGNMQASQELCDLMSARRSNVGHKEPRNPNCQGSCKSQ
SPATA31A4        --------------------------------------------------------------------------------
SPATA31A5  1021  SETQPQVCAAVVLLPDGQASVVPHASENLVSQVPQGHLQSMPTGNMRASQELHDLMAARRSKLVHEEPRNPNCQGSCKSQ
SPATA31A7  1021  SETQPQVCAAVVLLPDGQASVVPHASENLVSQVPQGHLQSMPTGNMRASQELHDLMAARRSKLVHEEPRNPNCQGSCKSQ
SPATA31A3  1021  SETQPQVCAAVVLLPDGQASVVPHASENLVSQVPQGHLQSMPTGNMRASQELHDLMAARRSKLVHEEPRKPNCQGSCKSQ
SPata31A2  1021  SETQPQVCAAVVLLPDGQASVVPHASENLVSQVPQGHLQSMPTGNMRASQELHDLMAARRSKLVHEEPRNPNCQGSCKNQ
SPATA31A6  1017  SETQPQVCAAVVLLPDGQASVVPHASENLVSQVPQGHLQSMPTGNMRASQELHDLMAARRSKLVQEEPRNPNCQGSCKSQ
spata31     680  LAGQPQIYTTTVLPPKRSRSMP------------------LPVDTLTSHVLGDIVVADMDNSLVQQRPSTPKHLVSQKSQ
```

```
SPATA31C1  953 SPMFPPTHKRENSRKPNLEKHEEMFQGLRTPQLTPGRKTEDTRQNEGVQLLPSKKQPPSISHFGENIKQFFETIFSKKER
SPATA31C2  947 SPMFPPTHKRENSRKPNLEKHEEMFQGLRTPQLTPGRKTEDTRQNEGVQLLPSKKQPPSISHFGENIKQFFQTIFSKKER
SPATA31A4      --------------------------------------------------------------------------------
SPATA31A5 1101 RPMFPPIHKSEKSRKPNLEKHEERLEGLRTPQLTPVRKTEDTHQDEGVQLLPSKKQPPSVSPFGENIKQIFQWIFSKKKS
SPATA31A7 1101 RPMFPPIHKSEKSRKPNLEKHEERLEGLRTPQLTPVRKTEDTHQDEGVQLLPSKKQPPSVSPFGENIKQIFQWIFSKKKS
SPATA31A3 1101 RPMFPPIHKSEKFRKPNLEKHEERLEGLRTPQLTPVRKTEDTHQDEGVQLLPSKKQPPSVSPFGENIKQIFQWIFSKKKS
SPata31A2 1101 RPMFPPIHKSEKSRKPNLEKHEERLEGLRTPQLTPVRKTEDTHQDEGVQLLPSKKQPPSVSHFGGNIKQFFQWIFSKKKS
SPATA31A6 1097 RPMFPPIHKSEKSRKPNLEKHEERLEGLRTPQLTPVRKTEDTHQDEGVQLLPSKKQPPSVSHFGENIKQFFQWIFSKKKS
spata31    742 IKMLAPTYQSEGTKRQSEIKYED------RPKLTPVTEKKANFGSQYYQTLPKIAQVLPGRPPQRHLGRFLQWIHPKKTI
```

PCNA Interacting motif IXXFF

Homing endonucleases

```
SPATA31C1 1033 K-----------PAPVTAESQKTVKNRSCVYGSSAEAERLMTAVGQIPEENMSLCHARHASKVNQQRQQFQAPVCGFPCN
SPATA31C2 1027 K-----------PAPVTAESQKTVKNRSCVYGSSAEAERLMTAVGQILEENMSLCHARHASKVNQQRQQFQAPVCGFPCN
SPATA31A4      --------------------------------------------------------------------------------
SPATA31A5 1181 K-----------PAPVTAESQKTVKNRSRVYSSSAEAQGLMTAVGQMLDEKMSLCHARHASKVNQHKQKFQAPVCGFPCN
SPATA31A7 1181 K-----------PAPVTAESQKTVKNRSRVYSSSAEAQGLMTAVGQMLDEKMSLCHARHASKVNQHKQKFQAPVCGFPCN
SPATA31A3 1181 K-----------PAPVTAESQKTVKNRSCVYSSSAEAQGLMTAVGQMLDEKMSLCHARHASKVNQHKQKFQAPVCGFPCN
SPata31A2 1181 K-----------PAPVTAESQKTVKNRSCVYSSSAEAQGLMTAVGQMLDEKMSLCHARHASKVNQHKQKFQVPVCGFPCN
SPATA31A6 1177 K-----------PAPVTAESQKTVKNRSCVYSSSAEAQGLMTAVGQMLDKKMSLCHAHHASKVNQHKQKFQAPVCGFPCN
spata31    816 KGHEFHPLKGNPTAAIVQNQRRQVRKIPYMDNNVTEAQELMTTVGQMLEKKMMLQHQPYASKFNQHREVPPAPMSRFSHG
```

Homing endonucleases

```
SPATA31C1 1102 HRHPFYSDHSRMLSYAASSQQATLKNQSRPNRDRQIRDQQPLKSVRCNNEQWGLRHPQLLLPKKAVSP----------VS
SPATA31C2 1096 HRHPFYSEHSRMLSYAASSQQATLKNQSRPNRDRQIRDQ-----------------------------------------
SPATA31A4      --------------------------------------------------------------------------------
SPATA31A5 1250 HRHLFYSEHGRILSYAASSQQATLKSQGCPNRDRQIRNQQPLKSVRCNNEQWGLRHPQILHPKKAVSP----------VS
SPATA31A7 1250 HRHLFYSEHGRILSYAASSQQATLKSQGCPNRDRQIRNQQPLKSVRCNNEQWGLRHPQILHPKKAVSP----------VS
SPATA31A3 1250 HRHLFYSEHGRILSYAASSQQATLKSQGCPNRDRQIRNQQPLKSVRCNNEQWGLRHPQILHPKKAVSP----------VS
SPata31A2 1250 HRHLFYSEHGRILSYAASSQQATLKSQGCPNRDRQIRNQQPLKSVRCNNEQWGLRHPQILHPKKAVSP----------VS
SPATA31A6 1246 HRHLFYSEHGRILSYAASSQQATLKSQGCPNRDRQIRNQQPLKSVRCNNEQWGLRHPQILHPKKAVSP----------VS
spata31    896 HMPVSYLQQRRAPSYPGSCS-----CQRCSVQNRHIRNQLPQRSVRFSKKPQNPRNPSHKKPQNPRNPSRLSRNAALNLV

SPATA31C1 1172 PPQHRPKTPSASSHHHH---------------------------
SPATA31C2      --------------------------------------------
SPATA31A4      --------------------------------------------
SPATA31A5 1320 PPQHWPKTSGASSHHHHCPRHCLLWEGI----------------
SPATA31A7 1320 PPQHWPKTSGASSHHHHCPRHCLLWEGI----------------
SPATA31A3 1320 PPQHWPKTSGASSHHHHCPRHCLLWEGI----------------
SPata31A2 1320 PLQHWPKTSGASSHHHHCPRHCLLWEGI----------------
SPATA31A6 1316 PPQHWPKTSGASSHHHHCPRHCLLWEGI----------------
spata31    971 NSQNRTIVPGNSNHHLYCPRHCALQRDVCRELGHSSVVFPNRKT
```
