# Supplementary material for: Segmental duplications and evolutionary acquisition of UV damage response in the SPATA31 gene family of primates and humans
Source: BMC Genomics. 2017 Mar 6;18:222. doi: 10.1186/s12864-017-3595-8 (PMC5338094; doi:10.1186/s12864-017-3595-8)
Supplement: Additional file 6: — Alignment of SPATA31 protein with CRY2 and EBV-BPLF1 proteins. The figure shows the alignment of SPATA31A1 (Q5TZJ5-1) protein with CRY2 (Q49AN0-1) (Top) and ENV-BPLF1 (P03186) (bottom) proteins. Similar (gray) and identical (black) amino acids are highlighted by using boxshade (http://www.ch.embnet.org/software/BOX_form.html). Alignment was performed by using Clustal-w and manually edited and only the region showing highest homology/similarity region is shown (starting from the amino acid position 166 of SPATA31A1) [31]. The overall calculated identity and similartiy between SPATA31A1 and CRY2 protein within the given window of alignment is 15% and 23%, and between SPATA31A1 and EBV-BPLF1 protein is 17% and 28%, respectively. Identity and similarity was calculated by GeneDoc software (version 2.7.000). (PDF 53 kb) [file 12864_2017_3595_MOESM6_ESM.pdf]

```
CRY2        1     MAATVATAAAVAPAPAPGTDSASSVHWFR---KGLRL----HDNPALLAAV-RGARCVRC----------
SPATA31A1   166   PQAKHPQDLASTPSPGPMTTSVSSLSASQPPEPSLPLEHPSPEPPALFPHPPHTPDPLACSPPPPKGFTA

CRY2        53    -----VYILDPWFAASSSVGINRWRFLLQSLEDLDTSLRKLNSRLFVVRGQPADVFPRLFKEWGVTRL-T
SPATA31A1   237   PPLRDSTLITPSHCDSVALPLGTVPQSLSPHEDLVASVPAIS----GLGGSNSH--VSASSRWQETARTS

CRY2        117   FEYDS------------EPFGKERDAAIMK-M--AKEAGVEVVTENSHTLYDLDRIIELNGQKPPLTYKR
SPATA31A1   307   CAFNSSVQQDHLSRHPPETYQMEAGSLFLLSSDGQNAVGIQVTETAKVNIWEEKEN--------------

CRY2        172   FQAIISRMELPKKPVGLVTSQQMESCRAEIQENHDETYGVPS------LEELGFPTEGLGPAVWQGGETE
SPATA31A1   377   VGSFTDRMT-PEKHLNSL-----RNLAKSLDAEQDTTNPKPFWNMGENSKQLPGPQKLSDPRLWQESFWK

CRY2        236   ALARL--------DKHLERKAWVANYERPRMNANSLLASPTGLSPYLRFGCLSCRLFYYRLWDLYKKVKR
SPATA31A1   447   NYSQLFWGLPSLHSESLVANAWVTDRSYTLQSPPFLFNEMSNVCPIQRETTMSPLLFQAQPPSH---LGP


EBV-BPLF1 451 VPPHRPPSAARLPPPVIPIPHQSPPASPTPHPAPVSTIAPSVTPSPRLPLQIPIPLPQAAPSNPKIPLTTPSPSPTAAAAPTTTTLSPPP
SPATA31A1 166 -------------PQAKHPQDLASTPSPGPMTTSVSSLSASQPPEPSLPLEHPSPEPPALFPHP-----PHTPDPLACSPPPPKGFTAPP

EBV-BPLF1 541 TQQQPPQSAAPAPSPLLPQQQPTPSAAPAPSPLLPQQQPPPSAARAPSPLPPQQQPLPSATPAPPPAQQLPPSATTLEPEKNHPPAADRA
SPATA31A1 256 LRDSTLITPSHCDSVALPLG------------------------TVPQSLSPHEDLVASVP-----------AISGLGGSNSHVSASSRW

EBV-BPLF1 631 GTEISPSPPFGQQPSFGDDASGGSGLVRYLSDLEEPFLSMSDSEEAESDLASDIPTTEDEDMFEDEVFSNSLESGSSAPTSPITLDTARS
SPATA31A1 356 QETARTSCAFN--SSVQQDHLSRHPPETYQMEAGSLFLLSSD---GQNAVGIQVTETAKVNIWEEKENVGSFT-----------------
```
