# Supplementary material for: Segmental duplications and evolutionary acquisition of UV damage response in the SPATA31 gene family of primates and humans
Source: BMC Genomics. 2017 Mar 6;18:222. doi: 10.1186/s12864-017-3595-8 (PMC5338094; doi:10.1186/s12864-017-3595-8)
Supplement: Additional file 14: — Frame Shift Mutation analysis. The upper table provides the number of reads with frameshifts per number of reads for the different annotated SPATA31 genes, the lower table provides the respective percentages. (PDF 44 kb) [file 12864_2017_3595_MOESM14_ESM.pdf]

Frameshift Mutation Analysis

	SPATA31C1-1	*SPATA31C1*	*SPATA31C2-1*	*SPATA31C2*	*SPATA31A1*	*SPATA31A3*	*SPATA31P2*	*SPATA31A5*	*SPATA31A6*	*SPATA31A7*	*SPATA31AP1*
**S5_(Low)**	12/30903	16/30931	359/28274	358/27914	12/22324	9/21948	524/62580	13/22093	18/20432	16/22647	7/8751
**S7_(Low)**	99/35340	90/35289	85/32191	83/31606	26/24198	37/23763	167/67780	50/23439	62/23442	48/24061	9/9200
**S12_(Low)**	123/37605	112/37311	183/34490	187/33947	103/25339	92/24772	325/70136	105/24663	11/23231	85/25284	78/9274
**S6_(High)**	21570/26914	21469/26801	14815/35443	14489/34864	9790/10496	9728/10440	34352/68058	9463/10147	2920/3233	9769/16734	259/346
**S8_(High)**	22362/24230	22461/24344	20085/21982	19680/21539	15582/16656	15559/16600	43539/45862	15480/16514	16392/17683	15730/16737	6924/7456
**S11_(High)**	23062/27683	23197/27697	34547/36518	33921/35943	18255/25123	17871/24590	66494/71169	17967/24664	1601/7756	18419/25330	11887/13175

Each Box Represents **(Deleted Read Counts/Total number of Read Counts)**

Please note that deleted read counts are based on the single base pair deletion at the 5th position from the PAM sequence where the most of the mutations observed. There are additional mutations elsewhere

Percentage of frameshift mutation

	SPATA31C1-1	*SPATA31C1*	*SPATA31C2-1*	*SPATA31C2*	*SPATA31A1*	*SPATA31A3*	*SPATA31P2*	*SPATA31A5*	*SPATA31A6*	*SPATA31A7*	*SPATA31AP1*
**S5_(Cl1)_(Low)**	*0.00*	*0.00*	*0.01*	*0.01*	*0.00*	*0.00*	*0.01*	*0.00*	*0.00*	*0.00*	*0.00*
**S7_(Low)**	*0.00*	*0.00*	*0.00*	*0.00*	*0.00*	*0.00*	*0.00*	*0.00*	*0.00*	*0.00*	*0.00*
**S12_(Low)**	*0.00*	*0.00*	*0.01*	*0.01*	*0.00*	*0.00*	*0.00*	*0.00*	*0.00*	*0.00*	*0.01*
**S6_(Cl2)_(High)**	*0.80*	*0.80*	*0.42*	*0.42*	*0.93*	*0.93*	*0.50*	*0.93*	*0.90*	*0.58*	*0.75*
**S8_(High)**	*0.92*	*0.92*	*0.91*	*0.91*	*0.94*	*0.94*	*0.95*	*0.94*	*0.93*	*0.94*	*0.93*
**S11_(High)**	*0.83*	*0.84*	*0.95*	*0.94*	*0.73*	*0.73*	*0.93*	*0.73*	*0.21*	*0.73*	*0.90*

e.
