# Supplementary material for: Segmental duplications and evolutionary acquisition of UV damage response in the SPATA31 gene family of primates and humans
Source: BMC Genomics. 2017 Mar 6;18:222. doi: 10.1186/s12864-017-3595-8 (PMC5338094; doi:10.1186/s12864-017-3595-8)
Supplement: Additional file 15: — List of CNVs of SPATA31 genes in individuals from Chinese, Finnish, Yoruba and Kenyan populations (PDF 88 kb) [file 12864_2017_3595_MOESM15_ESM.pdf]

Copy numbers and the numbers of genomic variants of the individuals from four populations

Ethnicity	Sample	CNV	CNV_CORRECTED
Chinese	HG00449	4.5	5
Chinese	HG00452	4.8	5
Chinese	HG00478	4.85	5
Chinese	HG00530	4.87	5
Chinese	HG00608	5.3	5
Finnish	HG00181	5.5	6
Chinese	HG00559	5.54	6
Kenyan	GM19396	5.6	6
Chinese	HG00614	5.6	6
Finnish	HG00268	5.7	6
Chinese	HG00475	5.7	6
Chinese	HG00531	5.7	6
Kenyan	GM19311	5.8	6
Chinese	HG00525	5.8	6
Chinese	HG00443	5.8	6
Chinese	HG00437	5.9	6
Yoruban	GM19192	6	6
Kenyan	GM19307	6	6
Finnish	HG00320	6	6
Finnish	HG00358	6	6
Chinese	HG00557	6	6
Chinese	HG00590	6	6
Yoruban	GM19159	6.1	6
Chinese	HG00406	6.1	6
Finnish	HG00285	6.2	6
Chinese	HG00512	6.2	6
Chinese	HG00473	6.2	6
Chinese	HG00445	6.2	6
Chinese	HG00464	6.2	6
Yoruban	GM18856	6.3	6
Yoruban	GM19101	6.3	6
Kenyan	GM19332	6.3	6
Finnish	HG00313	6.3	6
Chinese	HG00476	6.3	6
Chinese	HG00463	6.3	6
Yoruban	GM18881	6.4	6
Kenyan	GM19328	6.4	6
Finnish	HG00174	6.4	6
Finnish	HG00280	6.4	6
Finnish	HG00331	6.4	6
Finnish	HG00186	6.5	7
Finnish	HG00342	6.5	7
Chinese	HG00448	6.5	7
Chinese	HG00451	6.5	7
Chinese	HG00458	6.5	7
Chinese	HG00533	6.5	7
Yoruban	GM18852	6.6	7
Yoruban	GM18855	6.6	7
Yoruban	GM18908	6.6	7
Yoruban	GM19128	6.6	7
Yoruban	GM19137	6.6	7
Yoruban	GM19141	6.6	7
Yoruban	GM19143	6.6	7
Kenyan	GM19393	6.6	7
Finnish	HG00378	6.6	7
Chinese	HG00421	6.6	7

Chinese	HG00442	6.6	7
Yoruban	GM18907	6.7	7
Yoruban	GM19146	6.7	7
Kenyan	GM19323	6.7	7
Kenyan	GM19431	6.7	7
Finnish	HG00274	6.7	7
Finnish	HG00312	6.7	7
Finnish	HG00321	6.7	7
Finnish	HG00355	6.7	7
Chinese	HG00407	6.7	7
Chinese	HG00446	6.7	7
Chinese	HG00656	6.7	7
Yoruban	GM18861	6.8	7
Yoruban	GM18873	6.8	7
Yoruban	GM19144	6.8	7
Kenyan	GM19019	6.8	7
Kenyan	GM19376	6.8	7
Kenyan	GM19398	6.8	7
Kenyan	GM19313	6.8	7
Finnish	HG00315	6.8	7
Finnish	HG00329	6.8	7
Finnish	HG00334	6.8	7
Chinese	HG00404	6.8	7
Chinese	HG00427	6.8	7
Chinese	HG00542	6.8	7
Chinese	HG00457	6.8	7
Yoruban	GM18501	6.9	7
Yoruban	GM19092	6.9	7
Yoruban	GM19108	6.9	7
Yoruban	GM19118	6.9	7
Kenyan	GM19030	6.9	7
Kenyan	GM19435	6.9	7
Finnish	HG00176	6.9	7
Chinese	HG00436	6.9	7
Chinese	HG00501	6.9	7
Chinese	HG00628	6.9	7
Yoruban	GM18507	7	7
Yoruban	GM18871	7	7
Yoruban	GM19150	7	7
Yoruban	GM19190	7	7
Kenyan	GM19399	7	7
Kenyan	GM19037	7	7
Finnish	HG00272	7	7
Chinese	HG00556	7	7
Chinese	HG00500	7	7
Yoruban	GM18502	7.1	7
Yoruban	GM19129	7.1	7
Yoruban	GM19171	7.1	7
Yoruban	GM19175	7.1	7
Kenyan	GM19020	7.1	7
Kenyan	GM19438	7.1	7
Kenyan	GM19378	7.1	7
Kenyan	GM19346	7.1	7
Finnish	HG00190	7.1	7
Finnish	HG00308	7.1	7
Finnish	HG00309	7.1	7
Finnish	HG00311	7.1	7
Finnish	HG00327	7.1	7
Finnish	HG00357	7.1	7
Yoruban	GM18517	7.2	7

Yoruban	GM18870	7.2	7
Yoruban	GM18909	7.2	7
Yoruban	GM18917	7.2	7
Yoruban	GM19096	7.2	7
Yoruban	GM19099	7.2	7
Yoruban	GM19119	7.2	7
Yoruban	GM19130	7.2	7
Yoruban	GM19153	7.2	7
Kenyan	GM19039	7.2	7
Kenyan	GM19331	7.2	7
Kenyan	GM19321	7.2	7
Finnish	HG00178	7.2	7
Finnish	HG00335	7.2	7
Finnish	HG00337	7.2	7
Finnish	HG00364	7.2	7
Finnish	HG00367	7.2	7
Finnish	HG00371	7.2	7
Finnish	HG00375	7.2	7
Chinese	HG00513	7.2	7
Chinese	HG00683	7.2	7
Yoruban	GM18874	7.3	7
Yoruban	GM18915	7.3	7
Yoruban	GM18916	7.3	7
Yoruban	GM19116	7.3	7
Yoruban	GM19172	7.3	7
Yoruban	GM19189	7.3	7
Kenyan	GM19334	7.3	7
Kenyan	GM19429	7.3	7
Kenyan	GM19031	7.3	7
Kenyan	GM19390	7.3	7
Kenyan	GM19440	7.3	7
Kenyan	GM19401	7.3	7
Kenyan	GM19316	7.3	7
Finnish	HG00179	7.3	7
Finnish	HG00270	7.3	7
Finnish	HG00281	7.3	7
Finnish	HG00323	7.3	7
Finnish	HG00338	7.3	7
Finnish	HG00349	7.3	7
Finnish	HG00353	7.3	7
Finnish	HG00356	7.3	7
Finnish	HG00372	7.3	7
Finnish	HG00373	7.3	7
Chinese	HG00595	7.3	7
Yoruban	GM19127	7.4	7
Yoruban	GM19193	7.4	7
Kenyan	GM19027	7.4	7
Kenyan	GM19404	7.4	7
Kenyan	GM19319	7.4	7
Kenyan	GM19044	7.4	7
Kenyan	GM19360	7.4	7
Kenyan	GM19439	7.4	7
Kenyan	GM19046	7.4	7
Finnish	HG00341	7.4	7
Finnish	HG00346	7.4	7
Finnish	HG00379	7.4	7
Chinese	HG00651	7.4	7
Yoruban	GM18519	7.5	8
Yoruban	GM19095	7.5	8
Kenyan	GM19022	7.5	8

Kenyan	GM19318	7.5	8
Kenyan	GM19347	7.5	8
Kenyan	GM19372	7.5	8
Kenyan	GM19327	7.5	8
Kenyan	GM19434	7.5	8
Finnish	HG00278	7.5	8
Finnish	HG00319	7.5	8
Finnish	HG00362	7.5	8
Chinese	HG00524	7.5	8
Chinese	HG00534	7.5	8
Chinese	HG00619	7.5	8
Chinese	HG00653	7.5	8
Chinese	HG00654	7.5	8
Chinese	HG00692	7.5	8
Yoruban	GM18504	7.6	8
Yoruban	GM18523	7.6	8
Yoruban	GM18933	7.6	8
Kenyan	GM19017	7.6	8
Kenyan	GM19383	7.6	8
Kenyan	GM19436	7.6	8
Kenyan	GM19310	7.6	8
Kenyan	GM19428	7.6	8
Kenyan	GM19380	7.6	8
Finnish	HG00183	7.6	8
Finnish	HG00345	7.6	8
Finnish	HG00360	7.6	8
Chinese	HG00543	7.6	8
Chinese	HG00629	7.6	8
Yoruban	GM18487	7.7	8
Yoruban	GM18862	7.7	8
Yoruban	GM18910	7.7	8
Yoruban	GM19098	7.7	8
Yoruban	GM19113	7.7	8
Kenyan	GM19023	7.7	8
Kenyan	GM19385	7.7	8
Kenyan	GM19397	7.7	8
Kenyan	GM19338	7.7	8
Kenyan	GM19035	7.7	8
Kenyan	GM19371	7.7	8
Kenyan	GM19432	7.7	8
Kenyan	GM19373	7.7	8
Kenyan	GM19443	7.7	8
Kenyan	GM19374	7.7	8
Finnish	HG00275	7.7	8
Finnish	HG00276	7.7	8
Chinese	HG00698	7.7	8
Yoruban	GM18488	7.8	8
Yoruban	GM18868	7.8	8
Yoruban	GM19093	7.8	8
Yoruban	GM19102	7.8	8
Yoruban	GM19117	7.8	8
Yoruban	GM19147	7.8	8
Yoruban	GM19152	7.8	8
Kenyan	GM19025	7.8	8
Kenyan	GM19377	7.8	8
Kenyan	GM19036	7.8	8
Kenyan	GM19314	7.8	8
Kenyan	GM19315	7.8	8
Finnish	HG00266	7.8	8
Finnish	HG00277	7.8	8

Finnish	HG00324	7.8	8
Finnish	HG00368	7.8	8
Finnish	HG00376	7.8	8
Chinese	HG00428	7.8	8
Chinese	HG00607	7.8	8
Yoruban	GM19107	7.9	8
Yoruban	GM19114	7.9	8
Yoruban	GM19140	7.9	8
Kenyan	GM19026	7.9	8
Kenyan	GM19375	7.9	8
Kenyan	GM19437	7.9	8
Kenyan	GM19043	7.9	8
Kenyan	GM19379	7.9	8
Kenyan	GM19324	7.9	8
Kenyan	GM19392	7.9	8
Kenyan	GM19403	7.9	8
Finnish	HG00273	7.9	8
Finnish	HG00267	7.9	8
Finnish	HG00185	7.9	8
Finnish	HG00284	7.9	8
Finnish	HG00306	7.9	8
Finnish	HG00343	7.9	8
Finnish	HG00365	7.9	8
Finnish	HG00366	7.9	8
Chinese	HG00634	7.9	8
Chinese	HG00657	7.9	8
Chinese	HG00671	7.9	8
Yoruban	GM18913	8	8
Yoruban	GM18924	8	8
Yoruban	GM19131	8	8
Yoruban	GM19138	8	8
Kenyan	GM19041	8	8
Kenyan	GM19045	8	8
Kenyan	GM19402	8	8
Kenyan	GM19038	8	8
Kenyan	GM19308	8	8
Kenyan	GM19351	8	8
Finnish	HG00344	8	8
Chinese	HG00537	8	8
Chinese	HG00611	8	8
Yoruban	GM18508	8.1	8
Yoruban	GM18853	8.1	8
Kenyan	GM19312	8.1	8
Kenyan	GM19382	8.1	8
Finnish	HG00177	8.1	8
Finnish	HG00187	8.1	8
Finnish	HG00318	8.1	8
Finnish	HG00361	8.1	8
Chinese	HG00626	8.1	8
Chinese	HG00663	8.1	8
Yoruban	GM18489	8.2	8
Yoruban	GM19160	8.2	8
Kenyan	GM19024	8.2	8
Kenyan	GM19394	8.2	8
Yoruban	GM18516	8.3	8
Yoruban	GM19121	8.3	8
Finnish	HG00328	8.3	8
Finnish	HG00369	8.3	8
Chinese	HG00592	8.3	8
Chinese	HG00635	8.3	8

Chinese	HG00702	8.3	8
Yoruban	GM19105	8.4	8
Kenyan	GM19352	8.4	8
Kenyan	GM19430	8.4	8
Chinese	HG00689	8.4	8
Yoruban	GM18498	8.5	9
Yoruban	GM18510	8.5	9
Yoruban	GM18520	8.5	9
Yoruban	GM18858	8.5	9
Yoruban	GM19149	8.5	9
Kenyan	GM19391	8.5	9
Kenyan	GM19441	8.5	9
Kenyan	GM19381	8.5	9
Finnish	HG00332	8.5	9
Chinese	HG00472	8.5	9
Chinese	HG00684	8.5	9
Yoruban	GM18505	8.6	9
Yoruban	GM18511	8.6	9
Yoruban	GM18859	8.6	9
Yoruban	GM19185	8.6	9
Kenyan	GM19384	8.6	9
Finnish	HG00188	8.6	9
Finnish	HG00326	8.6	9
Finnish	HG00350	8.6	9
Finnish	HG00377	8.6	9
Chinese	HG00662	8.6	9
Chinese	HG00693	8.6	9
Chinese	HG00699	8.6	9
Yoruban	GM19122	8.7	9
Kenyan	GM19355	8.7	9
Finnish	HG00351	8.7	9
Chinese	HG00690	8.7	9
Yoruban	GM18499	8.8	9
Yoruban	GM18877	8.8	9
Finnish	HG00173	8.8	9
Finnish	HG00182	8.8	9
Finnish	HG00189	8.8	9
Chinese	HG00610	8.8	9
Chinese	HG00620	8.8	9
Chinese	HG00650	8.8	9
Chinese	HG00672	8.8	9
Yoruban	GM18486	8.9	9
Kenyan	GM19317	8.9	9
Finnish	HG00180	8.9	9
Finnish	HG00339	8.9	9
Kenyan	GM19309	9	9
Finnish	HG00359	9	9
Finnish	HG00380	9	9
Yoruban	GM18867	9.1	9
Yoruban	GM18934	9.1	9
Kenyan	GM19359	9.1	9
Finnish	HG00282	9.1	9
Kenyan	GM19395	9.2	9
Kenyan	GM19350	9.2	9
Chinese	HG00701	9.2	9
Yoruban	GM18912	9.3	9
Kenyan	GM19028	9.3	9
Chinese	HG00593	9.3	9
Chinese	HG00613	9.3	9
Kenyan	GM19444	9.4	9

Yoruban	GM19184	9.5	10
Chinese	HG00589	9.5	10
Chinese	HG00596	9.5	10
Yoruban	GM18923	9.7	10
Finnish	HG00336	9.8	10
Finnish	HG00310	9.9	10
Finnish	HG00269	10.3	10
Chinese	HG00625	10.4	10
Yoruban	GM18522	10.8	11
Finnish	HG00271	11.4	11
