# Supplementary material for: Segmental duplications and evolutionary acquisition of UV damage response in the SPATA31 gene family of primates and humans
Source: BMC Genomics. 2017 Mar 6;18:222. doi: 10.1186/s12864-017-3595-8 (PMC5338094; doi:10.1186/s12864-017-3595-8)
Supplement: Additional file 16: — PCR conditions and Primer List. PCR was performed in 20 μL reactions composed of 0.8 μL of a 10 mM dilution of the forward primer and reverse primer, 10 μL of Roche (11636103001) PCR Master Mix. The following PCR conditions were used (A): 3 min at 95 °C, followed by 40 cycles at 95 °C for 30 s, 55 °C 30 s, and 72 °C for 30 s followed by 7 min at 72 °C. The following real-time PCR conditions (B) were used: 3 min at 95 °C, followed by 50 cycles at 95 °C for 15 s, 55 °C 20 s, and 72 °C for 20 s elongation cycle. (PDF 93 kb) [file 12864_2017_3595_MOESM16_ESM.pdf]

	Primer1	Sequence	Primer2
**RT-PCR**	FM_907bF	ACAGAAGACACCCGTCAGAATGAAG	FM_R
	FM_907aF	TTCCCAGTCCTATCTCCTGCTTTTC	
	FM_All_F	ACCACCTCAGTCTCCTCCCTAAGTG	FM_All_R
	mSpata_ex1_F	GACTGGGGCTCTACCACCTGTTACTTC	mSpata_ex3_R
	UBE1-F	GAAGATCATCCCAGCCATTG	UBE1-R
**5'RACE PCR**	5' Anc	GGCCACGCGTCGACTAGTACGGGIIGGGIIGGGII	FM_AR_2
	UAP	$(CUG)_4$GGCCACGCGTCGACTAGTAC	FM_AR1
	AUAP	GGCCACGCGTCGACTAGTAC	FM_AR1B
**Bacterial Exp Construct**	PGEX_EcoR1_FM_F	CCCGAATTCatgtttccccctattcacaag	PGEX_EcoR1_FM_R
**Real-Time PC**	FM_Ex_1F	CACATGGAGAATCTTCCCTTTCCTT	FM_Ex_2R
	FM_907bF	ACAGAAGACACCCGTCAGAATGAAG	FM_R
	FM_907aF	TTCCCAGTCCTATCTCCTGCTTTTC	
	FM_All_F	ACCACCTCAGTCTCCTCCCTAAGTG	FM_All_R
	GAPDH-F	ATGACAACTTTGGTATCGTGGAAGG	GAPDH-R
	UBE1-F	GAAGATCATCCCAGCCATTG	UBE1-R
**Digital PCR**	SPATA31_A_ddF	ACAAGTCACAGAAACAGCCAAGGTC	SPATA31_A_ddR
	EIF2C1_F	CTGCTAGCCATCAGACGTAAG	EIF2C1_F
	Albumin_F	TTGTGGGCTGTAATCATCGTCTAGG	Albumin_R
	**Probes**		
	EIF2C1_probe	5HEX/ACTTTGTTG/ZEN/GTGGAGAAGGGCTGA, 3IABkFQ,	
	Albumin_Probe	5'HEX, CCCACACAAATCTCTCCCTGGCATT, 3'BHQ2	
	SPATA31_A_Probe	5'FAM, gatcgaatgaccccagaaaagcact, 3'BHQ1	Please note that ther
CRISPR/Cas targeting			
FM_ex1_px330	HsSPATA31_ex1_r_px330	CACCgatatccaacacccatggtg	SPATA31_ex1_px330
FM_ex1_px260	HsSPATA31_ex1_r_px260	AAACaagtgaggaagatatccaacacccatggtgGT	SPATA31_ex1_px260

Sequence2	Length	PCR Conditions
GCAAAGTGACATGTTCTCCTCCAGT	258	A
	258	A
GTGGGTGAGGGAAAAGTGCAGGT		
TGCTGTCTTGTTTAGCACTTGATG	288	
TTGAGGGTCATCTCCTCACC	255	A
AGTTGTGAAAGCAGGTCCGAAG		
TCTTCCATAGGCTCATGAGAGGACT		
ACTTAGGGAGGAGACTGAGGTGGT		
CCCGAATTCtcaaggagtcctcaatccttc		
GCTCTCAGACTGTGGTTTTTCATCC	251	B
GCAAAGTGACATGTTCTCCTCCAGT	258	B
GTGGGTGAGGGAAAAGTGCAGGT		B
GAAATGAGCTTGACAAAGTGGTCGT	442	B
TTGAGGGTCATCTCCTCACC	255	B
CTGTTTCGAGTTCTCTCCCATGTTC		METHODS
AGGGAGGGAGGGAAAGATAG		METHODS
GCTGGTTCTCTTTCACTGACATCTGC		METHODS

re is a 5 nt mismatch at 5' from SPATA31A6 sequence but it works well in our digital PCR

AAACcaccatgggtgttggatatc		METHODS
TAAAACcaccatgggtgttggatatcttcctcactt		METHODS
